# Tissue-specific differences in the assembly of mitochondrial Complex I are revealed by a novel ENU mutation in ECSIT

**DOI:** 10.1093/cvr/cvad101

**Published:** 2023-07-03

**Authors:** Thomas Nicol, Sara Falcone, Andrew Blease, Pratik Vikhe, Gabriele Civiletto, Saleh Salman Omairi, Carlo Viscomi, Ketan Patel, Paul K Potter

**Affiliations:** Mammalian Genetics Unit, MRC Harwell Institute, Becquerel Avenue, Didcot, OX11 0RD, Oxfordshire, UK; BHF Centre of Research Excellence, Division of Cardiovascular Medicine, Radcliffe Department of Medicine, John Radcliffe Hospital, University of Oxford, Oxford, UK; Mammalian Genetics Unit, MRC Harwell Institute, Becquerel Avenue, Didcot, OX11 0RD, Oxfordshire, UK; Centre for Cellular and Molecular Physiology, University of Oxford, Oxford, UK; Mammalian Genetics Unit, MRC Harwell Institute, Becquerel Avenue, Didcot, OX11 0RD, Oxfordshire, UK; Mammalian Genetics Unit, MRC Harwell Institute, Becquerel Avenue, Didcot, OX11 0RD, Oxfordshire, UK; MRC Mitochondrial Biology Unit, University of Cambridge, Cambridge, UK; School of Biological Sciences, University of Reading, Reading, UK; MRC Mitochondrial Biology Unit, University of Cambridge, Cambridge, UK; School of Biological Sciences, University of Reading, Reading, UK; Mammalian Genetics Unit, MRC Harwell Institute, Becquerel Avenue, Didcot, OX11 0RD, Oxfordshire, UK; Department Biological and Medical Sciences, Faculty of Health and Life Sciences, Oxford Brookes University, Oxford, UK

**Keywords:** Cardiac, Mitochondria, Complex I, Heart

## Abstract

**Aims:**

Mitochondrial Complex I assembly (MCIA) is a multi-step process that necessitates the involvement of a variety of assembly factors and chaperones to ensure that the final active enzyme is correctly assembled. The role of the assembly factor evolutionarily conserved signalling intermediate in the toll (ECSIT) pathway was studied across various murine tissues to determine its role in this process and how this varied between tissues of varying energetic demands. We hypothesized that many of the known functions of ECSIT were unhindered by the introduction of an ENU-induced mutation, while its role in Complex I assembly was affected on a tissue-specific basis.

**Methods and results:**

Here, we describe a mutation in the MCIA factor ECSIT that reveals tissue-specific requirements for ECSIT in Complex I assembly. MCIA is a multi-step process dependent on assembly factors that organize and arrange the individual subunits, allowing for their incorporation into the complete enzyme complex. We have identified an ENU-induced mutation in ECSIT (N209I) that exhibits a profound effect on Complex I component expression and assembly in heart tissue, resulting in hypertrophic cardiomyopathy in the absence of other phenotypes. The dysfunction of Complex I appears to be cardiac specific, leading to a loss of mitochondrial output as measured by Seahorse extracellular flux and various biochemical assays in heart tissue, while mitochondria from other tissues were unaffected.

**Conclusions:**

These data suggest that the mechanisms underlying Complex I assembly and activity may have tissue-specific elements tailored to the specific demands of cells and tissues. Our data suggest that tissues with high-energy demands, such as the heart, may utilize assembly factors in different ways to low-energy tissues in order to improve mitochondrial output. These data have implications for the diagnosis and treatment of various disorders of mitochondrial function as well as cardiac hypertrophy with no identifiable underlying genetic cause.

## Graphical Abstract

**Graphical Abstract:**
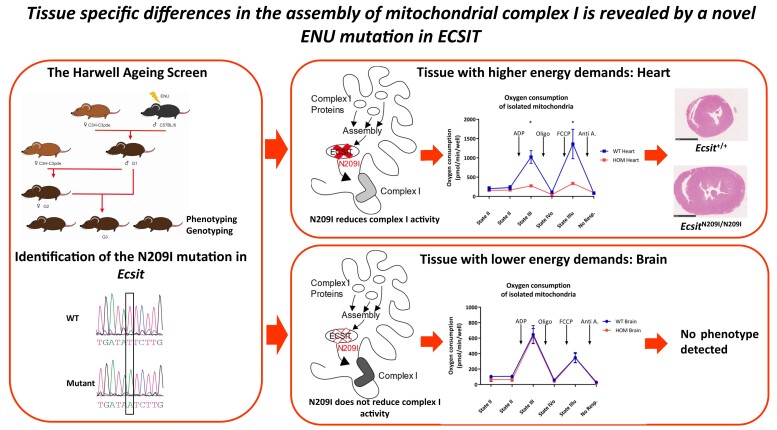

**Time of primary review: 28 days**

## 1. Introduction

Given the size, complexity, and contributions from the mitochondrial and nuclear genomes, it is unsurprising that the assembly of mitochondrial Complex I is an intricate process that we are only beginning to understand. Complex I consists of 45 subunits, 7 of which are encoded by the mitochondrial DNA and the remaining in the nucleus. Involved in its assembly are at least 14 assembly factors that are not thought to comprise part of the final structure but are essential in the intervening steps between isolated proteins and functional complex.^1,2^

The assembly process proceeds in a stepwise fashion, with individual building blocks or subassemblies forming first, before joining to form structural or functional portions of the complex and ultimately the complete complex. There are four main modules that must be assembled for full function: N, the NADH-binding domain; Q, the quinone-binding domain; and P_p_ and P_D_, the proximal and distal portions of the membrane arm involved in proton pumping.^3^

Among the assembly factors involved in Complex I assembly is the evolutionarily conserved signalling intermediate in the toll pathway (ECSIT). In humans, ECSIT is a 431 amino acid adapter protein with 2 identifiable isoforms (50/33 kDa) and a third potential isoform based on splice prediction (24 kDa).^4,5^

Human ECSIT protein has three recognizable domains in the full-length protein: an *N*-terminal mitochondrial-targeting sequence (amino acids 1–48), a highly ordered pentatricopeptide repeat (PPR) region (amino acids 90–266), and a less ordered C-terminal domain that shows some three-dimensional resemblance to pleckstrin homology domains (amino acids 275–380).^6^ Mouse ECSIT protein maintains roughly 73% sequence homology to the human protein, with the same mitochondrial-targeting sequence seen in the human protein (amino acids 1–48; results according to Phyre2 web server).^7^

ECSIT was first identified as interacting with the proteins TRAF6 (tumour necrosis factor receptor–associated factor 6) and MEKK-1/MAP3K1 (ERK kinase kinase-1/mitogen–activated protein kinase) in the Toll/interleukin-1 pathway. It has been shown that ECSIT binds to the multi-adapter protein TRAF6 and allows for the phosphorylation of MEKK1 (MAP3K1) into an active state. This phosphorylation event leads to activation of nuclear factor κB (NF-κB) and promotion of the innate immune response.^4^

During Complex I assembly, ECSIT interacts with NDUFAF1 and Complex I, and has previously been identified in large Complex I assembly complexes of ∼500, 600, and 850 kDa along with NDUFAF1. siRNA knock-down of ECSIT results in loss of Complex I protein levels and enzymatic activity.^6,8^ ACAD9 is another Complex I assembly factor that interacts with both ECSIT and NDUFAF1, and knock-down of ACAD9 leads to a decrease in NDUFAF1 and ECSIT protein levels and a reduction in functional Complex I levels.^9^ Together, these three proteins form part of the mitochondrial Complex I assembly (MCIA) complex along with TMEM126B and TIMMDC1.^6,10^ ACAD9 forms a homodimer that acts as the scaffold for the interaction of ECSIT and NDUFAF1, bringing the three proteins together. This trimer then interacts with the membrane bound proteins TMEM126B and TIMMDC1 before acting as part of the Complex I assembly process.^6^

Patients with Complex I deficiencies display a wide variety of phenotypes varying from syndromes, such as Leigh syndrome^11,12^ and MELAS syndrome^13,14^, which may arise in early childhood, to later onset or milder conditions. Among these, many other clinical features have been reported, including cases of exercise intolerance, renal tubular acidosis, lactic acidosis, cardiomyopathy, and encephalopathy due to mutations in Complex I subunits (NDUFV2 and NDUFS2)^15,16^ or assembly factors (ACAD9, NDUFAF1, and TMEM126B).^9,17–19^ Mutations in Complex I assembly factors result in a range of disease phenotypes^20^, which may suggest variability in the sensitivity of different tissues to deficiencies in Complex I activity.

Here, we describe a novel, ENU-induced mutation^21^ in the Complex I assembly factor ECSIT, which results in a profound cardiomyopathy phenotype with other tissues apparently unaffected. Our initial hypothesis was that this phenotype arose due to mitochondrial deficiency in the heart muscle, while other roles for ECSIT were likely unaffected. We demonstrate that this phenotype arises from an impairment of Complex I assembly in cardiac tissue, which is maintained at or close to normal levels in other tissues tested. Furthermore, the evidence suggests that ECSIT may have a tissue-specific role in the Complex I assembly process and could shed light on the differences in phenotypes observed in Complex I–deficient patients.

## 2. Methods

### 2.1 Mice

C57BL/6J and C3H-C3pde6b+ inbred mice were maintained in the Mary Lyon Centre in Harwell, UK, in specific pathogen-free conditions. All animal procedures were carried out under the guidance issued by the Medical Research Council in ‘Responsibility in the Use of Animals for Medical Research’ (July 1993) and in accordance with Home Office regulations (Home Office Project Licence No. 30/3070).

### 2.2 Generation of mutagenized mice

The original mutant mouse pedigree was derived from a G_3_ pedigree produced in the MRC Harwell ENU mutagenesis screen, as described previously.^21^ Briefly, male C57BL/6J mice were mutagenized with ENU and then mated to female C3H.Pde6b+ mice to generate G_1_ founder males, heterozygous for ENU-induced mutations. G_1_ males were subsequently bred to female C3H.Pde6b+ mice to generate G_2_ offspring. Lastly, G_2_ females were mated back to the original G_1_ founder to generate two G_3_ cohorts, both heterozygous and homozygous for ENU-induced mutations.

### 2.3 Mapping and next-generation sequencing

DNA from affected mice and littermate controls was tested on the Illumina Golden Gate ‘Mouse MD Linkage Panel’ (Oxford Genomics Centre, Wellcome Trust Centre for Human Genetics). DNA from the G_1_ founder of the pedigree was sent for whole-genome sequencing (WGS) employing the Illumina HiSeq platform (Oxford Genomics Centre, Wellcome Trust Centre for Human Genetics) and analysed as previously described.^21^ The *Ecsit* mutation was validated using Sanger Sequencing (Source: *Bioscience*).

### 2.4 Light and electron microscopy

For light microscopy, hearts fixed in 10% neutral-buffered formalin were embedded in paraffin wax and sectioned using a Finesse ME+ microtome (Thermo Fisher, Waltham, MA, USA). Transverse sections (T/S) were stained with haematoxylin and eosin.

For transmission electron microscopy (TEM), 1 mm^3^ cubes of left ventricular tissue were fixed in 3% glutaraldehyde and 4% formaldehyde in 0.1 M PIPES and post-fixed with 1% osmium tetroxide in 0.1 PIPES. Samples were taken from the left ventricular free wall of three wild-type and three *Ecsit^N209I/N209I^* males at 16 weeks of age. After serial dehydration in an increasing concentration of ethanol, the tissue was embedded in epoxy resin (TAAB) and polymerized overnight at 60°C. Golden ultrathin sections (70–80 nm) were cut with a diamond knife and collected on copper/palladium grids. To improve contrast, blocks were stained with 2% uranyl acetate and grids were stained with lead citrate.

Images were collected at the Wolfson bioimaging facility at the University of Bristol using a Tecnai 12 Biotwin electron microscope.

### 2.5 Echocardiography

Echocardiography was performed by the phenotyping core of the Mary Lyon centre at MRC Harwell. Twelve-week-old male and female mice were anaesthetized with 4% isoflurane (maintained at 1.5%) in oxygen and echocardiogram performed using a Vevo 770 high-resolution *in vivo* micro-imaging system with a Visualsonics RMV707B Probe (30 MHz). Body temperature was monitored using a rectal thermometer and maintained using a heat lamp at 36–38°C. Electrocardiogram (ECG) monitoring was performed using limb electrodes, and the heart rate was maintained at or above 400 b.p.m. Short-axis B- and M-mode images were taken using the papillary muscles as a point of reference for the positioning of the probe. Image contrast and gain functions were used for clarity and frame rate of 110 Hz used throughout. Measurements were taken from M-mode images using the inbuilt Vevo software.

### 2.6 Western blot

Proteins were extracted from tissue by homogenizing in RIPA buffer (150 mM NaCl, 1% NP-40, 0.5% DOC, 0.1% SDS, 50 mM Tris, pH 7.5), containing phosphatase and protease (Roche, Basel Switzerland) inhibitors in precellys CK28 homogenization tubes (Bertin Instruments, Montigny-le-Bretonneux, France).

Proteins were isolated from macrophages by scraping the macrophages from the plate and centrifuging to obtain the cell pellet before lysing in RIPA buffer containing phosphatase and protease inhibitors.

Protein concentration was measured by Bradford assay (Bio-Rad, Hercules, CA, USA) and on a µQuant plate reader (BioTek Instruments Inc., Winooski, VT, USA). Twenty micrograms of total protein were mixed with LDS sample buffer (Invitrogen, Waltham, MA, USA) and reducing agent (Invitrogen, Waltham, MA, USA) loaded onto NuPAGE™ 4–12% Bis-Tris protein gels (Invitrogen, Waltham, MA, USA). Electrophoresis was performed at 200 V for 60 min at room temperature in 1× MOPS.

PVDF membrane (GE) was activated in absolute methanol for 1 min and proteins were transferred using an X-Cell blot module (Invitrogen, Waltham, MA, USA) containing 1× transfer buffer (Invitrogen, Waltham, MA, USA), 20% methanol, and 1× antioxidant (Invitrogen, Waltham, MA, USA) according to the manufacturer’s instructions.

Following transfer, membranes are blocked in either 5% w/v milk powder in phosphate buffered saline (PBS) containing 0.1% tween or 5% bovine serum albumin (BSA) in TBS containing 0.1% tween (for phosphorylated proteins) for 60 min with shaking. Primary antibodies diluted in 5% milk/PBS-T or 5% BSA/TBS-T are incubated overnight at 4°C before three, 5 min washes in PBS-T or TBS-T. Secondary antibodies (Section 2.8) are diluted in 5% milk/PBS-T or 5% BSA/TBS-T and incubated for 1 h. Fluorescent secondary antibodies are protected from light during this and subsequent steps. Membranes are subsequently washed a further three times before drying. Blots were scanned using a LI-COR Odyssey Cl-x or SA scanner (LI-COR Biosciences, Cambridge, UK). Image Studio Lite software (LI-COR, Lincoln, NE, USA) is used for quantification and analysis (median, three pixel border background).

### 2.7 Primary antibodies

**Table:** 

Antibody (protein)	Species/clonality	Supplier	Code	Dilution	Size (kDa)
6X His	Rabbit poly	Origene, Rockville, MD, USA	TA150031	1:1000	N/A
ACAD9	Rabbit poly	Abcam, Cambridge, UK	ab99952	1:500	69
ATP5A	Mouse mono	Abcam, Cambridge, UK	ab14748	1:1000	53
CD5—BV421	Rat mono	Becton, Dickinson and Company, Franklin Lakes, NJ, USA	562739	1:800 (FACS)	N/A
CD11b—PE—CF594	Rat mono	Becton, Dickinson and Company, Franklin Lakes, NJ, USA	562317	1:200 (FACS)	N/A
CLPP	Rabbit poly	Abcam, Cambridge, UK	ab124822	1:5000	26
COXIV	Mouse mono	Abcam, Cambridge, UK	ab14744	1:1000	17
ECSIT	Rabbit poly	Abcam, Cambridge, UK	ab21288	1:1000	50/45
F4/80—PE	Rat mono	Thermo Fisher, Waltham, MA, USA	12-4801-80	1:200 (FACS)	N/A
JNK	Rabbit poly	CST	9252S	1:1000	54/46
LONP1	Rabbit poly	Abcam, Cambridge, UK	ab103809	1:1000	106
LY6C—FITC	Rat mono	Abcam, Cambridge, UK	ab15686	1:200 (FACS)	N/A
LY6G—BV421	Rat mono	Becton, Dickinson and Company, Franklin Lakes, NJ, USA	562737	1:200 (FACS)	N/A
MFN2	Mouse mono	Abcam, Cambridge, UK	ab56889	1:1000	86
MHCI	Mouse mono	DSHB, Iowa City, IA, USA	A4.840	1:1 (ICC)	N/A
MHCIIA	Mouse mono	DSHB, Iowa City, IA, USA	A4.74	1:1 (ICC)	N/A
MHCIIB	Mouse mono	DSHB, Iowa City, IA, USA	BF.F3	1:1 (ICC)	N/A
MHCIIX	Mouse mono	DSHB, Iowa City, IA, USA	6H1	1:1 (ICC)	N/A
MTCO1	Mouse mono	Abcam, Cambridge, UK	ab14705	1:2000	40
MT-ND1	Rabbit mono	Abcam, Cambridge, UK	ab181848	1:1000	36
Myc	Mouse mono	Origene, Rockville, MD, USA	TA150121	1:1000	N/A
NDUFA10	Rabbit poly	Abcam, Cambridge, UK	ab103026	1:500	41
NDUFA3	Mouse mono	Santa Cruz, Dallas, TX, USA	sc-365351-S	1:1000	9
NDUFA8	Mouse mono	Santa Cruz, Dallas, TX, USA	sc-398097-S	1:1000	20
NDUFB1	Rabbit poly	Abcam, Cambridge, UK	ab201302	1:1000	12
NDUFB11	Rabbit mono	Abcam, Cambridge, UK	ab183716	1:10 000	17
NDUFB3	Mouse mono	Santa Cruz, Dallas, TX, USA	sc-393351-S	1:1000	12
NDUFB8	Mouse mono	Abcam, Cambridge, UK	ab110242	1:2000	22
NDUFC2	Rabbit mono	Abcam, Cambridge, UK	ab192265	1:1000	14
NDUFS2	Rabbit mono	Abcam, Cambridge, UK	ab192022	1:1000	49
NDUFS3	Mouse mono	Santa Cruz, Dallas, TX, USA	sc-374282-S	1:1000	30
NDUFS8	Mouse mono	Santa Cruz, Dallas, TX, USA	sc-515527-S	1:1000	23
NDUFV2	Rabbit mono	Abcam, Cambridge, UK	ab183715	1:1000	24
NF-κB	Mouse mono	Cell Signaling Technology, Danvers, MA, USA	6956T	1:1000	65
OPA1	Rabbit poly	Abcam, Cambridge, UK	ab42364	1:1000	92/86
p38-MAPK	Rabbit poly	Cell Signaling Technology, Danvers, MA, USA	9212S	1:1000	43
PGC1α	Rabbit poly	Abcam, Cambridge, UK	ab54481	1:1000	105
Phospho JNK (T183/Y185)	Rabbit poly	Cell Signaling Technology, Danvers, MA, USA	9251S	1:1000	46/54
Phospho p38-MAPK (T180/Y182)	Mouse mono	Cell Signaling Technology, Danvers, MA, USA	9216S	1:2000	43
PINK1	Rabbit poly	Abcam, Cambridge, UK	ab23707	1:1000	66
SDHA	Mouse mono	Abcam, Cambridge, UK	ab14715	1:10 000	70
TOM20	Rabbit poly	Abcam, Cambridge, UK	ab199641	1:1000	16
UQCRC2	Mouse mono	Abcam, Cambridge, UK	ab14745	1:1000	49
VDAC	Rabbit poly	Abcam, Cambridge, UK	ab15895	1:1000	31
α-TUBULIN	Rabbit mono	Abcam, Cambridge, UK	ab176560	1:2000	50

### 2.8 Secondary antibodies

**Table:** 

Antibody	Supplier	Code	Dilution
IRDye® 680LT goat anti-mouse IgG (H + L)	Li-Cor, Lincoln, NE, USA	926-68020	1:15 000
IRDye® 680LT goat anti-rabbit IgG (H + L)	Li-Cor, Lincoln, NE, USA	926-68021	1:15 000
IRDye® 800CW goat anti-rabbit IgG (H + L	Li-Cor, Lincoln, NE, USA	926-32211	1:15 000
IRDye® 800CW goat anti-mouse IgG (H + L)	Li-Cor, Lincoln, NE, USA	926-32210	1:15 000
Alexa fluor 633 goat anti-mouse	Thermo Fisher, Waltham, MA, USA	A20146	1:200 (ICC)
Alexa fluor 488 goat anti-mouse	Thermo Fisher, Waltham, MA, USA	A11029	1:200 (ICC)
Anti-mouse HRP	Promega, Madison, WI, USA	W402B	1:1000
Anti-rabbit HRP	Promega, Madison, WI, USA	W401B	1:1000

### 2.9 Flow cytometry analysis

Immune cell profiling in blood was performed by flow cytometry as described previously.^22^ Samples were collected from 12-week-old male and female wild-type and *Ecsit^N209I/N209I^* animals. Briefly, blood was collected by retro-orbital bleeding in lithium heparin tubes, from the mice under terminal anaesthesia induced by an intraperitoneal overdose of sodium pentobarbital. For flow cytometry analysis, 50 µL of blood was resuspended in 1 mL of red blood cell (RBC) lysis buffer (BioLegend, San Diego, CA, USA) for 10 min on ice followed by two washes with 1 mL PBS. The final pellet was resuspended in FACS buffer [5 mM ethylenediaminetetraacetic acid (EDTA), 0.5% foetal calf serum in PBS] and transferred to V-bottom 96-well plate. The cell suspension was incubated for 15 min with CD16/CD32 antibody (Becton, Dickinson and Company, Franklin Lakes, NJ, USA) at a dilution of 1:100. After centrifugation at 800 *g* for 1 min, the cell pellets were resuspended in 100 μL of antibody cocktail: F4/80—PE (1:200; Thermo Fisher, Waltham, MA, USA), CD11b—PE—CF594 (1:200), Ly6G—BV421 (1:200), Ly6C—FITC (1:200) and CD5—BD421 (1:800) Becton, Dickinson and Company, Franklin Lakes, NJ, USA) and incubated for 20 min in the dark. Cells were centrifuged and washed twice with FACS buffer and finally fixed by adding 210 μL of 0.5% PFA. Flow cytometry was performed using a BD FACSCanto II system and FlowJo software (Becton, Dickinson and Company, Franklin Lakes, NJ, USA) was used to analyse the data obtained.

### 2.10 Macrophage culture

Twelve-week-old wild-type and *Ecsit^N209I/N209I^* animals were sacrificed by cervical dislocation and bone marrow flushed from femur and tibia with PBS containing 0.6 mM EDTA. Cell suspension was filtered through a pre-wetted 70 µm cell strainer. Cells were pelleted at 400 *g* for 7 min (4°C). RBCs were removed by resuspending the pellet in 3 mL of RBC lysis buffer (155 mM NH_4_Cl, 12 mM NaHCO_3_, 0.1 mM EDTA) and incubating for 1 min before diluting in 10 mL of PBS (0.6 mM EDTA) and again pelleted at 400 *g*.

Finally, cells were plated at a concentration of 2.5 × 10^6^ cells/mL in Dulbecco’s modified Eagle’s medium (DMEM; pyruvate, glutamine) containing 10% FBS, 100 U/mL penicillin–streptomycin and 100 ng/mL of macrophage colony-stimulating factor (MCSF; Cell Guidance Systems). Cells were maintained at 37°C, 5% CO_2_ with media changes on Days 3 and 6. On Day 7, cells were harvested by manual scraping with PBS (0.6 mM EDTA). Cells were counted again and re-plated at a concentration of 6 × 10^6^ cells/well of a six-well plate in DMEM (pyruvate, glutamine), 10% FBS, 100 U/mL penicillin–streptomycin, 100 ng/mL MCSF. Plated cells were activated with 100 ng/mL of lipopolysaccharide (LPS) added directly to the media and incubated for 24 h at 37°C, 5% CO_2_.

Activated cells were harvested by manual scraping with PBS (0.6 mM EDTA).

### 2.11 Cloning and transfection

Full-length *Ecsit* clone (Dharmacon MMM1013-202766953) was ligated into pCMV6-AC-HIS (Origene—PS10002) vector following incorporation of flanking restriction enzyme sites by PCR (*Sgf*I—AGGCGATCGCCATGAGCTGGGTGCAGGTCAACTT, Tm, 80.1°C, *Mlu*I—GCGACGCGTACTTTGCCCCTGCTGCTGCTCTG—Tm 72.0°C, expected amplicon size 1693 bp, MGI:1349469) and grown in XL-10 Gold *Escherichia coli* (Agilent). Site-directed mutagenesis (Q5-SDM—NEB) was utilized to introduce the N209I mutation (Forward—CGATTCAAGATTATCAACCCCTAC—Tm 62.1°C, Reverse—GGTGAACCACAGCTTCATC—Tm 61.5°C, expected amplicon size 7595 bp). Full-length Ndufaf1 (Dharmacon MMM1013-202762755) was similarly incorporated into pCMV6-Entry (Origene—PS10001, *Sgf*I—GAGGCGATCGCCATGTCTTCCATTCACAAATTACT—Tm 73.6°C, *Mlu*I—GCGACGCGTTCTGAAGAGTCTTGGGTTAAGAA—Tm 72.0°C, expected amplicon size 1472 bp, MGI:1916952). Vector DNA isolated by QIAprep Spin Miniprep kit (Qiagen).

pCMV6-AC-HIS-Ecsit (wild type or *Ecsit^N209I^*), pCMV6-Entry-TRAF6, pCMV6-Entry-ACAD9, and pCMV6-Entry-NDUFAF1 were transfected into Hek293T cells using jetPRIME reagent (Polyplus). Hek293T cells were plated at 2.5 × 10^5^ cells/well (six-well plate) in DMEM (high glucose, glutamax, 10% FBS, 100 U/mL penicillin–streptomycin).

### 2.12 Co-IP

Forty-eight hours of following transfection, HEK293T cells transfected with relevant vectors to express ECSIT or associated proteins were briefly washed with PBS and lysed in RIPA buffer (150 mM NaCl, 1% NP-40, 0.5% DOC, 0.1% SDS, 50 mM Tris, pH 7.5) with protease inhibitors (Roche, Basel, Switzerland). Protein concentration was assessed by Bradford assay (Bio-Rad, Hercules, CA, USA) and protein diluted to 1 mg/mL of protein lysate was pre-cleared with 20 µL of protein G sepharose bead slurry (Sigma) for 1 h to remove native immunoglobulins. Protein G beads were removed by briefly spinning at 1000 *g* for 1 min and the supernatant incubated with 4 µg of relevant antibody (outlined in key resources table) over night to bind the protein of interest. Following antibody binding, lysate was incubated with 20 µL of protein G sepharose bead slurry for 1 h to bind antibody and attached proteins. Beads were again pelleted by centrifugation at 100 *g* for 1 min and washed three times in RIPA buffer. Beads were left in 30 µL of RIPA buffer and 1× LDS sample buffer (Invitrogen) and reducing agent (Invitrogen) added before boiling the sample at 95°C for 10 min to dissociate the beads from the bound antibody.

Samples were loaded onto NuPAGE™ 4–12% Bis-Tris protein gels (Invitrogen) and run as with western blots.

### 2.13 Mitochondrial isolation

Mitochondria were isolated from frozen heart and brain tissue taken from wild-type and *Ecsit^N209I/N209I^* animals. Hearts were lysed in 10 mL/g of homogenization medium A (0.32 M sucrose, 1 mM EDTA, 10 mM tris-HCl, pH 7.4, filter sterilized) in an Elvehjem–Potter homogenizer on ice by hand. Homogenate was centrifuged at 1000 *g* or 5 min at 4°C and supernatant retained. Supernatant was centrifuged for 2 min at 9000 *g* at 4°C before removing the supernatant and fluffy coat, leaving behind the mitochondrial pellet. The mitochondrial pellet was subsequently resuspended in 100 µL of homogenization medium A and centrifuged at 9000 *g* for 10 min at 4°C, discarding the supernatant. This wash step is repeated five times, and the mitochondrial pellet is stored at −80°C. Brains were homogenized in the same fashion using 5 mL/g of homogenization medium AT (0.075 M sucrose, 0.225 M mannitol, 1 mM EGTA, 10 mM tris-HCl, pH 7.4, filter sterilized). Mitochondria were resuspended in 100 µL of homogenization medium and the concentration determined by Bradford assay (Bio-Rad, Hercules, CA, USA).

### 2.14 In gel activity

Fifty micrograms of isolated mitochondria were resuspended in 1× native sample buffer (Invitrogen) 2% digitonin and 1× protease inhibitors (Roche, Basel, Switzerland) and incubated for 1 h on ice before addition of 0.5% G-250 sample additive (Invitrogen). Prepared samples were run on NativePAGE™ 3–12% Bis-Tris gels under native conditions at 150 V for 30 min with 1× native cathode buffer (Invitrogen) followed by 90 min at 250 V in 0.1× native cathode buffer. The gel was then incubated for 1 h in 150 µM NADH, 3 mM nitro blue tetrazolium and 2 mM Tris-HCl (pH 7.4). Activity of Complex I corresponds to the depth of the blue-purple stain.

### 2.15 Seahorse

Mitochondria for seahorse analysis were isolated from freshly dissected hearts and brains obtained from wild-type and *Ecsit^N209I/N209I^* animals. Samples were kept on ice and homogenized in MSHE + BSA (70 mM sucrose, 210 mM mannitol, 5 mM HEPES, 1 mM EGTA, 0.2% w/v FFA-free BSA, 1 M KOH) in a Dounce homogenizer using both A and B pestles. Mitochondria were separated from the lysate by subsequent centrifugation at 800 *g* for 10 min at 4°C twice, discarding the pellet after each step. The supernatant is retained and centrifuged at 8000 *g* for 10 min at 4°C. The remaining mitochondrial pellet is suspended in MSHE + BSA and the concentration measured using a Bradford assay (Bio-Rad, Hercules, CA, USA). Isolated mitochondria are diluted 1:10 in ice cold mitochondrial assay solution (MAS) (70 mM sucrose, 220 mM mannitol, 10 mM KH_2_PO_4_, 5 mM MgCl_2_, 2 mM HEPES, 1 mM EGTA, 0.2% w/v FFA-free BSA, 1 M KOH) before diluting further in MAS to the desired concentration and loaded onto Seahorse XF24 plates at a concentration of 5 µg/well. The plate is centrifuged at 2000 *g* for 20 min at 4°C to ensure mitochondria are adherent.

Four hundred and fifty microlitres of MAS containing 10 mM pyruvate and 10 mM malate are added to the mitochondria in the plate, and the plate is incubated at 37°C for 10 min without CO_2_. Measurements are made on the seahorse with the addition of 40 mM ADP (4 mM final), 20 µM oligomycin (2 µM final), 40 µM FCCP, and anti-mycin A (4 µM final). Measurements were taken over a period 3 min flanked by 1 min mix periods.

### 2.16 Blue-native PAGE

Two hundred and fifty micrograms of isolated mitochondria were resuspended in 1× native sample buffer (Invitrogen) 2% digitonin and 1× protease inhibitors (Roche, Basel, Switzerland) and incubated for 1 h on ice, centrifuged at 20 000 *g* for 30 min at 4°C before the supernatant was removed and addition of 0.5% G-250 sample additive (Invitrogen). First-dimensional blue-native PAGE (BN-PAGE) was run on NativePAGE™ 3–12% Bis-Tris gels under native conditions at 150 V for 30 min with 1× native cathode buffer (Invitrogen) followed by 90 min at 250 V in 0.1× native cathode buffer. Second-dimensional BN-PAGE, first-dimensional lanes are cut from the gel and incubated in 1% SDS with 1% beta-mercaptoethanol for 1 h before the gel slice is loaded into a NuPAGE 4–13% 1.0 mm × two-dimensional (2D) gel (Invitrogen). Gels were run at 200 V for 50 min at room temperature in MOPS. Transfer for PVDF membrane and remaining steps were performed as with western blot.

### 2.17 Muscle fibre typing

Soleus and extensor digitorum longus (EDL) from male wild-type and *Ecsit^N209I/N209I^* animals were frozen over isopropanol on liquid nitrogen. Following freezing, frozen muscle samples were mounted in Tissue Tech freezing medium (Jung) and cooled by dry ice/ethanol. Cryosections taken at 10 µM thick were dried for 30 min at room temperature before being washed three times in PBS and incubated in a permeabilization buffer solution (4 mM HEPES, 3 mM MgCl_2_, 10 mM NaCl, 1.5 mM sodium azide, 60 mM sucrose, 0.1% Triton X-100) for 15 min. Following permeabilization, samples were washed in wash buffer [1× PBS with 5% foetal calf serum (v/v) and 0.05% Triton X-100] for 30 min at room temperature.

Primary antibodies against MHCI, MHCIIA, MCHIIX, and MHCIIB were diluted in wash buffer and incubated overnight at 4°C. The next day, samples are incubated in secondary antibody diluted in wash buffer for 1 h in darkness. Finally, slides were mounted in fluorescent mounting medium and myonuclei visualized with 2.5 µg/mL DAPI.

Fluorescence microscopy was performed with Zeiss Axio Imagerr AI, and images were captured with an Axiocam digital camera. Analysis was performed with Zeiss Axiovision computer software version 4.8.

### 2.18 Statistical analysis

All statistical analyses were carried out using Microsoft Excel and GraphPad Prism 8 (GraphPad Inc.). All analysis is displayed as mean ± standard error of the mean (SEM). Shapiro–Wilk and/or Kolmogorov–Smirnov tests were used to test for normality, depending on sample size. Where a normal distribution was observed, Student’s *t*-test was used where a single variable accounts for differences in two groups and one-way analysis of variance (ANOVA) with Tukey’s multiple comparisons test for multiple groups separated by a single variable. Where a non-normal distribution was observed, Student’s *t*-test with Welch’s correction was applied or in the case of multiple group comparisons, Friedman’s test with Dunn’s multiple comparison test. Differences were considered significant at *P*-values of <0.05. *P < 0.05, **P < 0.01, ***P < 0.001, ****P < 0.0001.

## 3. Results

### 3.1 Phenotype identification and genetic characterization

As part of a phenotype-driven screen to identify mutations resulting in age-related and chronic disease, we identified a group of related mice,^21^ exhibiting various signs of ill health (sudden weight loss, hunched appearance, piloerect coat, inactivity) or that died unexpectedly. Post-mortem analysis revealed enlarged hearts in these mice, and histological analysis showed characteristic signs of hypertrophic cardiomyopathy (HCM): enlargement and disorganization of the cardiomyocytes and the presence of vacuolation (see Supplementary material online, *Figure S1*).

**Figure 1:**
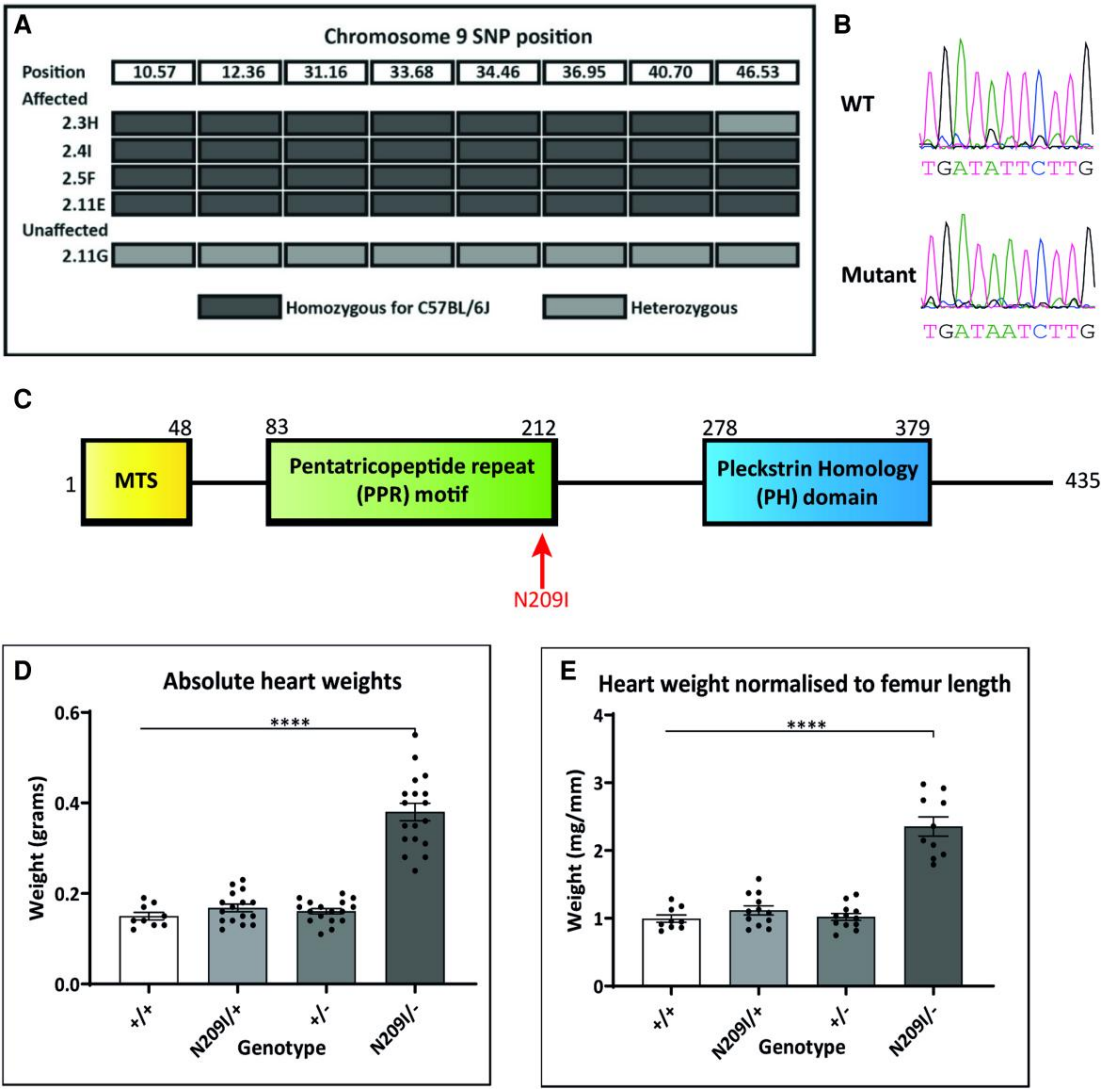
(*A*) Chromosome 9 mapping location identified by SNP mapping. All affected animals are homozygous for SNPs derived from the C57BL/6J founder between the proximal end and 46.53 Mb. (*B*) Sanger sequencing confirmation of an A to T transversion at Position 916 of *Ecsit*. (*C*) Heart weights from wild type (*n* = 8), heterozygous N209I mutant (*n* = 16), heterozygous knockout (*n* = 16), and compound heterozygote animals (*n* = 17) confirm that the loss of ECSIT protein function is causative of the hypertrophy phenotype observed. (*D*) Absolute and (*E*) Normalized heart weights from wild type, heterozygous N209I mutant, heterozygous knockout, and compound heterozygote animals. One-way ANOVA with Tukey’s multiple comparisons. Mean ± SEM, ****P < 0.0001.

The causative mutation was mapped to a 46 Mb region at the proximal end of Chromosome 9 (*Figure 1A*). WGS identified a single coding variant within the mapping region, an A to T transversion at nucleotide 916 of the gene *Ecsit*. This mutation was validated by Sanger sequencing (*Figure 1B*) and shown to lie in the predicted PPR motif of the mouse ECSIT protein (*Figure 1C*; protein domains predicted by Phyre 2 webserver.^7^) *Ecsit^N209I/+^* mice were crossed to *Ecsit^+/−^* animals to produce the compound heterozygotes and other intermediate genotypes. Heart weights from the four genotypes produced (*Figure 1D* and *E*) demonstrate that the phenotype is only present in compound heterozygotes, thus confirming that the mutation in *Ecsit* is the causative allele.

### 3.2 Cardiac phenotyping

**Figure 2:**
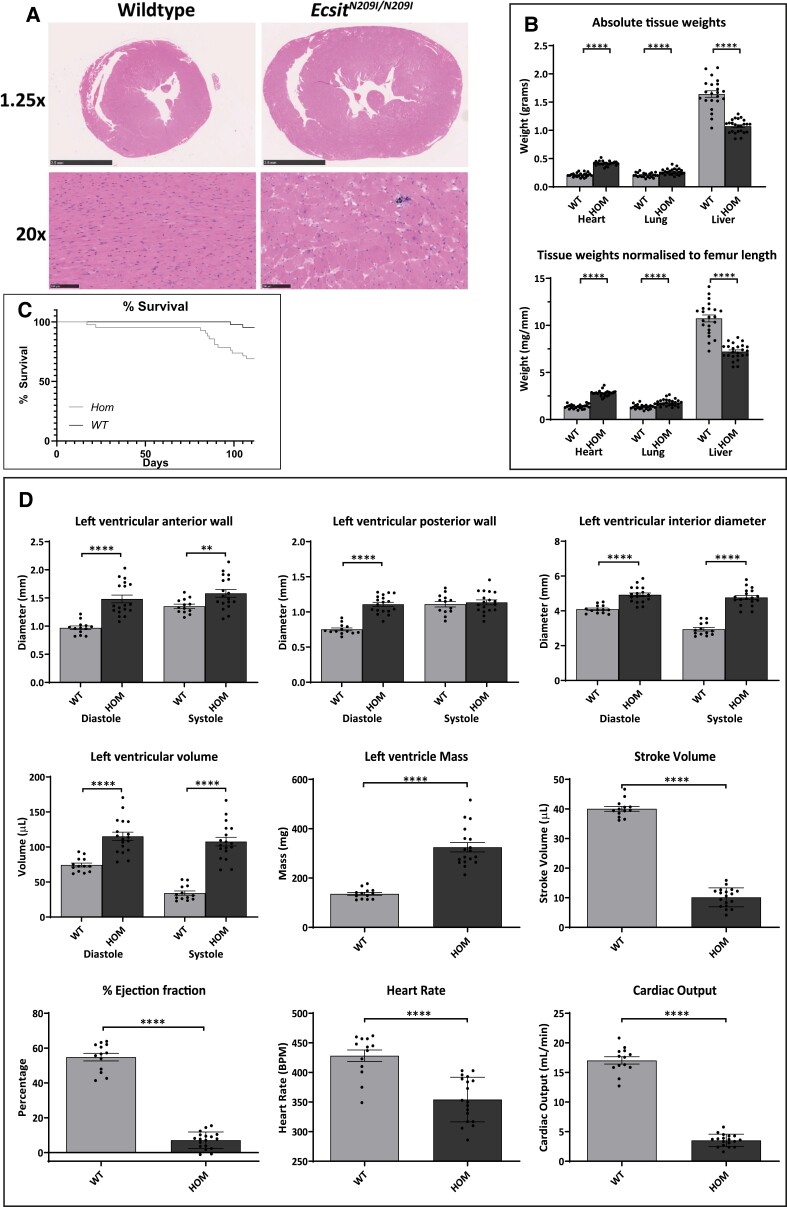
(*A*) Histology of wild-type and *Ecsit^N209I/N209I^* (*n* = 23) hearts demonstrating overall size increase as well as disorganized, enlarged, and vacuolated cardiomyocytes, indicative of HCM. (*B*) Absolute and normalized (to femur length) weights of heart, lung, and liver in backcrossed animals demonstrating phenotype is still present and that congestion is primarily of the left heart (*n* = 22 and 24, unpaired *t*-test). (*C*) Kaplan–Meier survival plot of wild-type and *Ecsit^N209I/N209I^* animals up to 12 weeks of age demonstrating a significant mortality in mutant animals (∼30%) by 12 weeks of age. (*D*) M-mode measurements of echocardiography showing significant enlargement of anterior and posterior wall as well as an increase in ventricular volume and mass. Furthermore, key measurements of cardiac function, stroke volume, ejection fraction, and cardiac output are significantly reduced (*n* = 13 and 18, unpaired *t*-test OR unpaired *t*-test with Welch’s correction—LVAW;d, LVvol;s, LVmass). Mean ± SEM, **P < 0.01, ****P < 0.0001.

The mutant line was backcrossed to C3H.Pde6b+ for five generations to produce an incipient congenic line used for all further phenotype characterization. Hearts from wild-type and *Ecsit^N209I/N209I^* animals were collected as a time course (birth, 1, 2, 4, 6, 8, and 12 weeks; *Figure 2A* and Supplementary material online, *Figure S2*), to determine the onset of disease and characterize the progression. Signs of HCM (vacuolation, mineralization, myocyte disorganization) were present from 6 weeks of age with progression (myocyte hypertrophy) clear at 8 and 12 weeks of age. In addition, tissue weights (*Figure 2B*), both in absolute values and normalized to body weight or femur length, demonstrated a significant increase in the heart weight of *Ecsit^N209I/N209I^* animals compared with controls at 12 weeks of age. Lung weights also showed a significant increase in *Ecsit^N209I/N209I^* animals, suggesting lung congestion resulting from left ventricular hypertrophy, conversely, and liver weights was significantly reduced in *Ecsit^N209I/N209I^* animals, while this was not investigated further, it does suggest that there may be alterations to the overall metabolic profile of these animals.

Echocardiography was performed on animals at 12 weeks of age (*Figure 2C* and Supplementary material online, *Figure S3*), demonstrating a thickening of the left ventricular anterior and posterior walls coupled with an overall increase in left ventricular mass and volume. Stroke volume, ejection fraction, and cardiac output all demonstrate a profound reduction in *Ecsit^N209I/N209I^* animals in comparison with wild type. Taken together, these data confirm the presence of HCM and suggest that there may also be some dilation of the left ventricle.

No difference was seen in ECG parameters (data not shown) or in muscle fibre types of the soleus and EDL muscles of the hind limb (see Supplementary material online, *Figure S4A* and *B*). Muscle fibre analysis did reveal a significant reduction in cross-sectional area of both the soleus and EDL in *Ecsit^N209I/N209I^* animals in comparison with wild type (see Supplementary material online, *Figure S4C* and *D*), although it is unclear if this is a result of the general reduction in size of the mutant animals or a true muscle phenotype.

Taken together, these data confirm that an HCM phenotype in the *Ecsit^N209I/N209I^* animals in comparison with wild type. This phenotype is present in the absence of other profound muscular phenotypes, although it may be involved in the development of overall body mass and fat reserves.

### 3.3 Identification of the pathogenic pathway underlying the HCM

ECSIT has been shown previously to be involved in both the Toll-like receptor (TLR) response^4,23^ and in the assembly of Complex I of the mitochondrial electron transport chain,^6,8^ both of which could be considered as causes for the development of an HCM phenotype. To determine which pathway was the underlying driver for the development of this phenotype, characterization of both the TLR response and mitochondrial electron transport assembly was undertaken.

**Figure 3:**
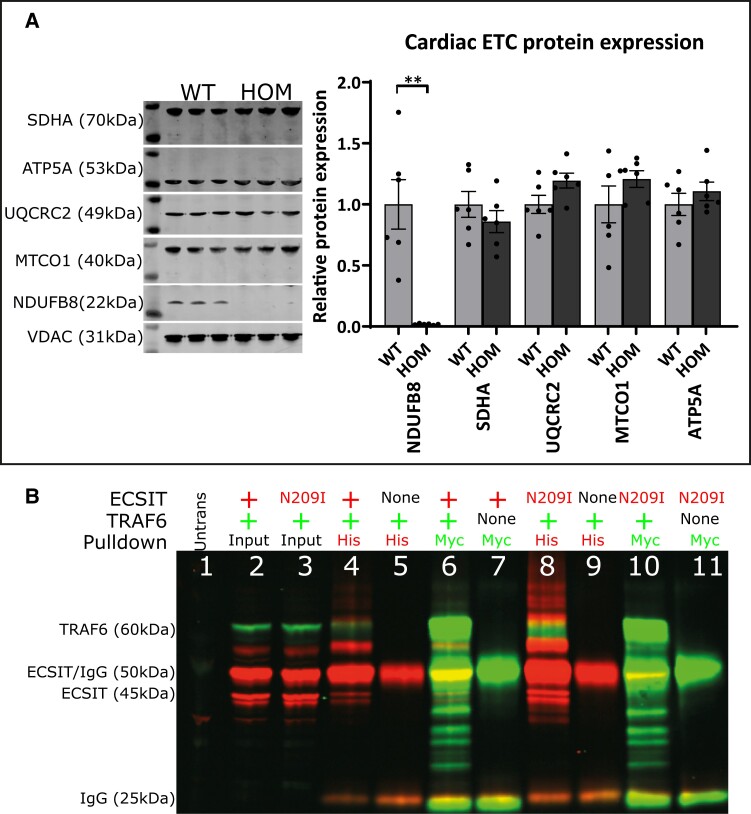
(*A*) Representative cardiac electron transport chain protein levels for each complex (I—NDUFB8, II—SDHA, III—UQCRC2, IV—MTCO1, V—ATP5A) (*n* = 6, unpaired *t*-test). (*B*) Immunoprecipitation of wild-type and mutant ECSIT (His tagged; 45 and 50 kDa) with full-length wild-type TRAF6 (Myc tagged; 60 kDa; green: Myc) and rabbit (red: His). (1) Untransfected input lysate, (2) wild-type ECSIT(His) + wild-type TRAF6(Myc) input lysate, (3) ECSIT N209I(His) + wild-type TRAF6(Myc) input lysate, (4) wild-type ECSIT(His) + wild-type TRAF6(Myc) anti-His immunoprecipitation, (5) empty AC-His vector + wild-type TRAF6(Myc) anti-His immunoprecipitation, (6) wild-type ECSIT(His) + wild-type TRAF6(Myc) anti-Myc immunoprecipitation, (7) wild-type ECSIT(His) + empty entry(Myc) vector anti-Myc immunoprecipitation, (8) N209I ECSIT(His) + wild-type TRAF6(Myc) anti-His immunoprecipitation, (9) Empty AC-His vector + wild-type TRAF6(Myc) anti-His immunoprecipitation, (10) N209I ECSIT(His) + wild-type TRAF6(Myc) anti-Myc immunoprecipitation, (11) N209I ECSIT + empty entry(Myc) vector anti-Myc immunoprecipitation (representative of *n* = 3 experimental repeats). Mean ± SEM, **P < 0.01.

Mitochondrial electron transport chain proteins NDUFB8 (CI), SDHA (CII), UQCRC2 (CIII), MTCO1 (CIV), and ATP5A (CV) were assessed in whole-heart lysate from male and female animals at 16 weeks of age by western blot. A 98% reduction (*P* = 0.0046) was present in Complex I protein levels in the heart (*Figure 3A*). No differences were observed in any other electron transport chain proteins. In addition, Complex I protein levels were also reduced in brain (∼30%), kidney (∼30%), and liver (∼60%) (see Supplementary material online, *Figure S5*).

To assess the role of the TLR response in the development of the cardiomyopathy phenotype FACS analysis on whole blood from 12-week-old male and female animals was performed to determine the constituents of the total leucocyte fraction. Results demonstrate a small reduction in the percentage of circulating lymphocytes and macrophages with no differences in monocyte or neutrophil levels (see Supplementary material online, *Figure S6A*).

In addition, bone marrow–derived macrophages (BMDMs) were cultured from the bone marrow of 12-week-old *Ecsit^N209I/N209I^* and *Ecsit^+/+^* animals. Following stimulation with LPS, the levels and phosphorylation of p38-MAPK and JNK were determined by western blot. Results show no significant alterations in the TLR response to stimulation with LPS between *Ecsit^+/+^* and *Ecsit^N209I/N209I^* BMDMs, indicating that the N209I mutation of the ECSIT protein does not significantly affect the function of this protein in signal transduction through the TLR pathway (see Supplementary material online, *Figure S6B* and *C*).

Finally, the interaction between ECSIT and TRAF6 protein was determined by co-immunoprecipitation. 6xHis-tagged ECSIT (pCMV6-AC-HIS-ECSIT or pCMV6-AC-HIS-ECSIT^N209I^) and Myc-tagged TRAF6 (pCMV6-Entry-TRAF6) were overexpressed in HEK293T cells, and cell lysate was collected for co-immunoprecipitation with antibodies against the relevant tags. Results (*Figure 3B*) confirm the interaction between both 50 and 45 kDa wild-type ECSIT and 60 kDA TRAF6 proteins. Furthermore, this interaction is maintained in the presence of the N209I mutation in the ECSIT protein, indicating that the N209I mutation does not deleteriously affect the function of the ECSIT protein in this protein–protein interaction.

Taken together, these data indicate that the TLR function of the ECSIT protein is not significantly affected by the mutation of ECSIT; however, the assembly of the electron transport chain Supercomplex I is significantly impacted by this mutation and this effect is most profound in the heart tissue.

### 3.4 A cardiac mitochondrial defect

**Figure 4:**
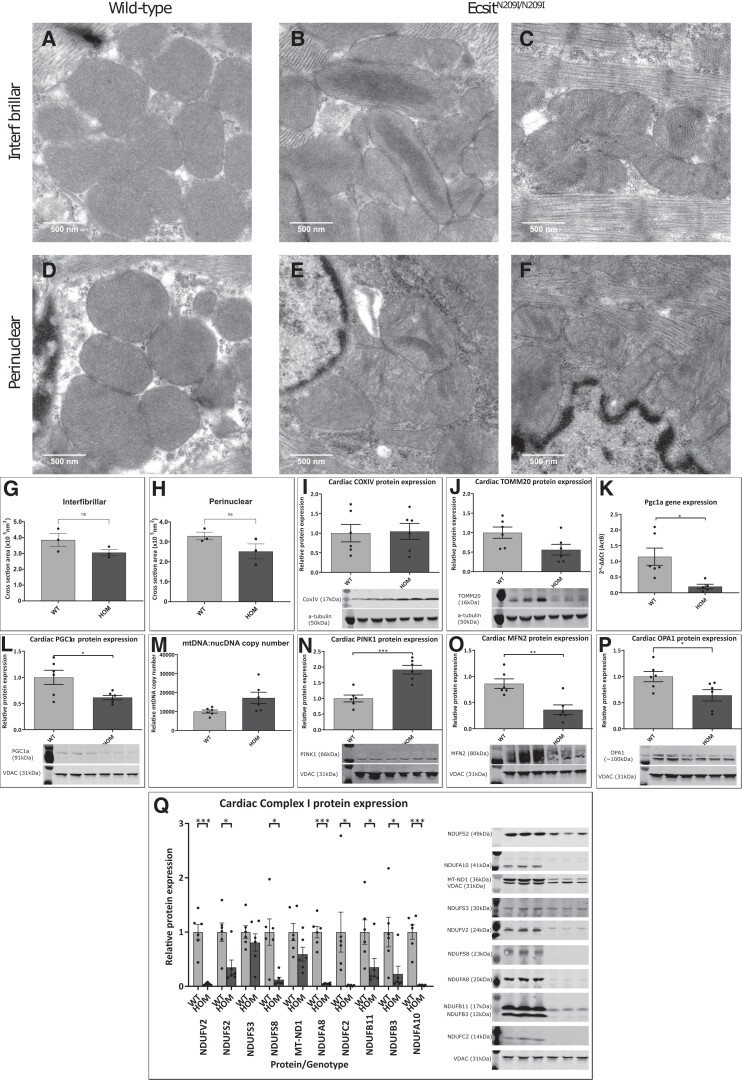
Representative TEM images of wild-type and *Ecsit^N209I/N209I^* interfibrillar and perinuclear mitochondria from cardiac tissue demonstrating structural abnormalities observed in *Ecsit^N209I/N209I^* samples (*n* = 3). Wild-type interfibrillar (*A*) and perinuclear (*B*) mitochondria demonstrate consistent evenly stacked cristae with no signs of disorganization. Mutant interfibrillar mitochondria demonstrate phenotypes such as hyper condensed (*C*) and disorganized cristae (*D*). Perinuclear mitochondria from mutant hearts also demonstrate disorganization (*E* and *F*), although hyper-packed cristae were not observed. Scale bars = 500 nm. (*G* and *H*) Measurements of mitochondria demonstrate a significant reduction in cross-sectional area of both interfibrillar and perinuclear mitochondria. (*I–L*) Normalized cardiac COXIV, TOMM20 and PGC1α protein, and mRNA expression demonstrating no differences in inner or outer membrane protein abundance but a decrease of mitochondrial biogenesis pathways at both the mRNA and protein level (*n* = 6, unpaired *t*-test). (*M*) Quantification of mitochondrial and nuclear DNA demonstrate a significant increase in relative amount of mtDNA in *Ecsit^N209I/N209I^* heart muscle in comparison with controls (*N–P*) quantification of PINK1 demonstrating an increase in protein levels typically associated with increased mitochondrial degradation and of mitochondrial fusion proteins, OPA1 and MFN2, typically associated with alterations to mitochondrial dynamics and quality control. (*Q*) Cardiac Complex I protein abundance of various proteins representing Complex I subunits and accessory proteins, N (V2), Q (S2, S3, S8), PP (ND1, A8, C2), PD (B11, B3), and the accessory protein NDUFA10. Quantification (normalized to VDAC, relative to wild-type average) shows a significant reduction in protein levels of all except two proteins, NDUFS3 and MT-ND1 (*n* = 6, unpaired *t*-test). Mean ± SEM, *P < 0.05, **P < 0.01, ***P < 0.001.

Mitochondrial structure was assessed in the hearts of 16-week-old animals by TEM (*Figure 4A–F* and Supplementary material online, *Figure S7A–D*). Micrographs demonstrate that, while there was no swelling of mitochondria or significant changes in mitochondrial size, a small but non-significant reduction in mitochondrial cross-sectional area was observed (*Figure 4G* and *H*) in *Ecsit^N209I/N209I^* animals, and there were signs of hypercondensed and disorganized cristae in both interfibrillar and perinuclear mitochondria, suggestive of mitochondrial dysfunction. Western blot analysis of the inner mitochondrial membrane protein COXIV showed no difference between wild-type and *Ecsit^N209I/N209I^* hearts (*Figure 4I*). However, the outer mitochondrial membrane protein TOMM20 showed a marked reduction in protein levels, although they did not achieve statistical significance (*Figure 4J*). Additionally, the master regulator of mitochondrial biogenesis, PGC1α, was significantly reduced at both the mRNA (20%, *P* = 0.0136) and protein level (29.5%, *P* = 0.0363) in *Ecsit^N209I/N209I^* hearts (*Figure 4K* and *L*), indicating that there is a dysregulation of the mitochondrial biogenesis pathway. To further investigate this result, qPCR of mitochondrial DNA was performed to determine mitochondrial DNA copy number in cardiac tissue relative to nuclear DNA; this demonstrated a significant increase in mtDNA in *Ecsit^N209I/N209I^* hearts in comparison with wild-type controls (*Figure 4P*). These data show a mixed result, with some metrics indicative of a loss of mitochondrial mass or biogenesis, while others (mt:nDNA ratio) indicate an increase.

To understand the relationship between mitochondrial biogenesis and degradation, we investigated whether the mutation in *Ecsit* was affecting mitochondrial dynamics through alteration of the PINK1/Parkin pathway or fusion/fission proteins. These results demonstrated a significant reduction in both MFN2 and OPA1, both mitochondrial fusion proteins (*Figure 4N* and *O*) and a significant increase in PINK1 (*Figure 4P*) in the heart tissue of *Ecsit^N209I/N209I^* animals. Functionally, these data are suggestive of a loss of mitochondrial mass and function with classical regulatory proteins (PGC1α), fusion/fission (MFN2 and OPA1), and degradation proteins (PINK1) all altered in a direction that suggests mitochondria are undergoing degradation. Taken together, these data suggest that there may be a tendency for increased mitochondrial turnover and a disturbance in overall mitochondrial dynamics.

Complex I is the largest of the respiratory chain complexes and has multiple distinct domains, each with a dedicated function, subassembly process, and associated assembly factors. Western blot (*Figure 4Q*) of proteins from each of these subassemblies (N—NDUFV2, Q—NDUFS2, NDUFS3, and NDUFS8, P_p_—NDUFC2, NDUFA8, and MT-ND1, P_D_—NDUFB11 and NDUFB3) as well as accessory subunit (NDUFA10) demonstrate a reduction of protein in each of the subunits as result of the N209I mutation in ECSIT in 16-week-old animals. ECSIT was previously demonstrated to be involved early in the assembly of the *P*_P_ arm of Complex I as part of the MCIA complex,^6,10^ and these results confirm that loss of this essential step has a downstream effect on the continued assembly of the complex.

Assessment of mRNA expression levels of these proteins further demonstrates that this failure to assemble intact Complex I is due to a loss of protein abundance as well as expression (see Supplementary material online, *Figure S8*). All proteins in each of the subassemblies of Complex I (N—NDUFV2, Q—NDUFS2, P_p_—NDUFC2, P_D_—NDUFB11, NDUFB8) demonstrate a loss of expression at the mRNA level as well as a reduction in protein abundance.

To confirm whether loss of mitochondrial protein levels was causative of, or secondary to the cardiac hypertrophy phenotype observed, further samples were taken from 2-week-old animals before the onset of any cardiac phenotype. Assessment of these samples against the same panel of proteins used in *Figure 3* (see Supplementary material online, *Figure S9*) demonstrates that mitochondrial Complex I protein (Ndufb8) is significantly reduced at this early time, while other mitochondrial proteins are unchanged, as was seen at the later time point.

### 3.5 Tissue specificity of the mitochondrial defect

**Figure 5:**
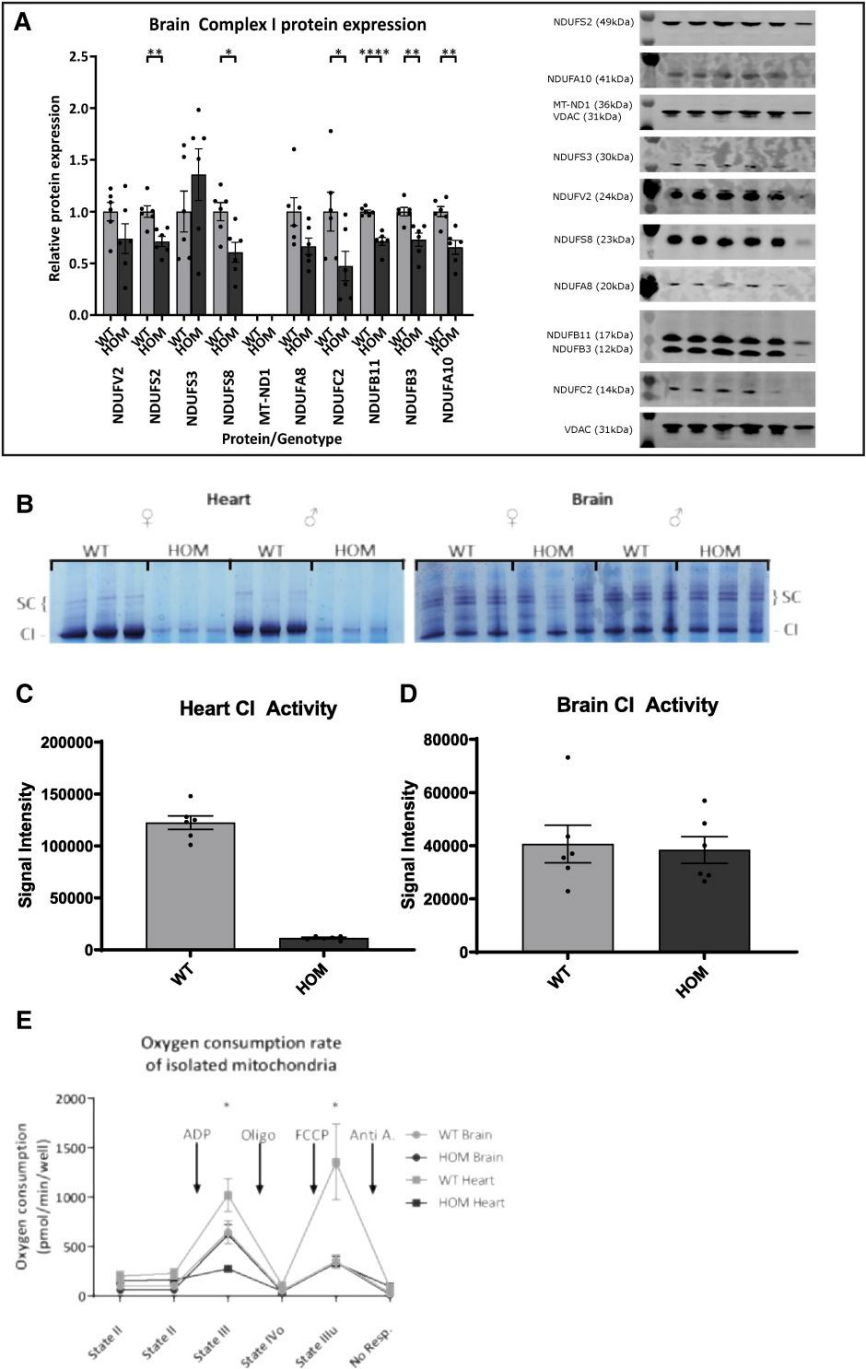
(*A*) Brain Complex I protein abundance of various proteins representing Complex I subunits and accessory proteins, N (V2), Q (S2, S3, S8), PP (ND1, A8, C2), PD (B11, B3), and the accessory protein NDUFA10. Quantification (normalized to VDAC, relative to WT average) shows a significant reduction in protein levels of all except four proteins, NDUFV2, NDUFS3, MT-ND1, and NDUFA8. MT-ND1 was not detectable by western blot in brain samples (*n* = 6, unpaired *t*-test). (*B*) In gel activity assay of Complex I in mitochondria isolated from heart and brain tissue of wild-type and *Ecsit^N209I/N209I^* animals. Depth of stain corresponds to Complex I activity with deeper staining reflecting greater activity. Results show a reduction in Complex I activity of *Ecsit^N209I/N209I^* hearts, while brains show no differences between genotypes (*n* = 6). (*C*) Quantification and statistical analysis of Complex I in gel activity in cardiac mitochondria. Analysis confirms the significant reduction in Complex I activity in cardiac mitochondria (*n* = 6, unpaired *t*-test). (*D*) Quantification and statistical analysis of Complex I in gel activity in brain mitochondria. Analysis shows no differences between mitochondria from wild-type and *Ecsit^N209I/N209I^* brains (*n* = 6, unpaired *t*-test). (*E*) Seahorse oxygen consumption rate measurements from wild-type and *Ecsit^N209I/N209I^* heart and brain mitochondria. Significant differences can be seen between wild-type and *Ecsit^N209I/N209I^* heart mitochondria during State III and State IIIu respiration. No differences are seen between wild-type and *Ecsit^N209I/N209I^* brain mitochondria (*n* = 4, RM one-way ANOVA with Tukey’s multiple comparisons OR Friedman’s test with Dunn’s multiple comparison for State III). Mean ± SEM, *P < 0.05, **P < 0.01, ****P < 0.0001.

To investigate the underlying cause of why no other severe phenotypes were observed as part of the normal screening process,^21^ western blots of various Complex I proteins were also performed in brain tissue taken from the same 16-week-old animals used for cardiac protein assessment (*Figure 5A*). These results show a significant reduction several Complex I proteins, although less severe than in heart lysate. In particular, Complex I protein levels were reduced in brain (∼30%), kidney (∼30%), and liver (∼60%), and not significantly altered in skeletal muscle (see Supplementary material online, *Figure S5*). Gel activity assays (*Figure 5B* and *C*) confirmed a reduction in activity of Complex I in isolated mitochondria from heart muscle, while no observable difference in activity levels could be seen in mitochondria isolated from brain. Mitochondria from liver also showed a small reduction in Complex I activity, but taken with other data, this does not seem to represent a significant loss of Complex I function as seen in cardiac mitochondria (see Supplementary material online, *Figure S5E*). This suggests that the reduction seen in Complex I protein levels in brain and liver was insufficient to reduce the activity of the complete enzyme complex. These results were verified by seahorse extracellular flux assay performed in the presence of pyruvate and malate on isolated mitochondria from heart and brain tissue of wild-type and *Ecsit^N209I/N209I^* animals (*Figure 5D*). This confirmed a significant reduction in State III and State IIIu oxygen consumption levels in isolated heart mitochondria from *Ecsit^N209I/N209I^* animals with no changes seen in isolated brain mitochondria from the same animals.

Taken together, these data suggest that while ECSIT is ubiquitously expressed, its role in Complex I assembly may differ between tissues, resulting in a less severe mitochondrial deficiency in certain tissues.

### 3.6 ECSIT protein levels and interactions

**Figure 6:**
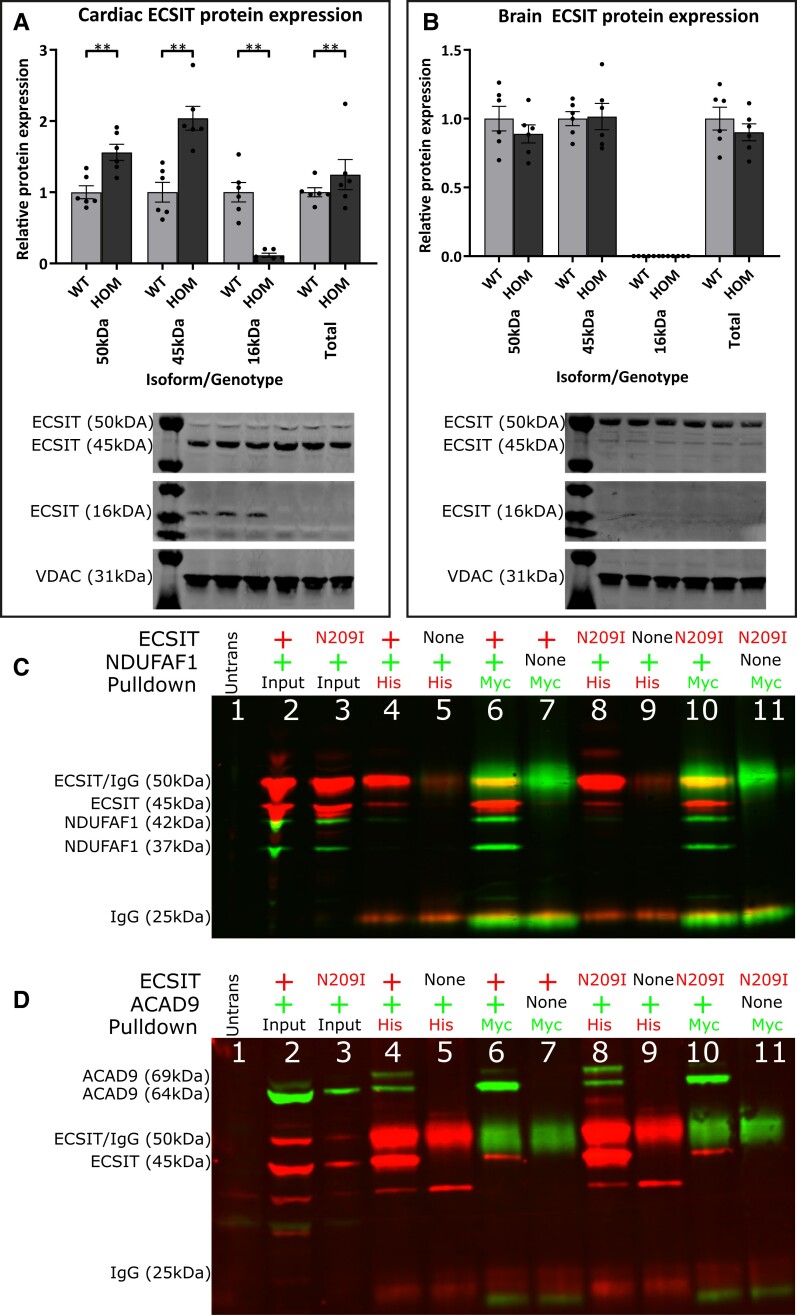
(*A* and *B*) Cardiac and brain ECSIT protein abundance normalized to loading control VDAC and demonstrated as fold change in comparison with wild-type control. Results show an increase in both 50 and 45 kDa ECSIT protein in *Ecsit^N209I/N209I^* animals compared with wild types (*n* = 6, unpaired *t*-test). In contrast, 16 kDa ECSIT demonstrates a significant reduction in *Ecsit^N209I/N209I^* animals, to the point where it is essentially undetectable in cardiac tissue from these animals. (*C* and *D*) Immunoprecipitation of wild-type and mutant ECSIT (His tagged; 45 and 50 kDa) with full-length wild-type NDUFAF1 or ACAD9 (Myc tagged; 60 kDa; green: Myc, red: His). (1) untransfected input lysate, (2) wild-type ECSIT(His) + wild-type NDUFAF1/ACAD9(Myc) input lysate, (3) ECSIT N209I(His) + wild-type NDUFAF1/ACAD9(Myc) input lysate, (4) wild-type ECSIT(His) + wild-type NDUFAF1/ACAD9(Myc) anti-His immunoprecipitation, (5) empty AC-His vector + wild-type NDUFAF1/ACAD9(Myc) anti-His immunoprecipitation, (6) wild-type ECSIT(His) + wild-type NDUFAF1/ACAD9(Myc) anti-Myc immunoprecipitation, (7) wild-type ECSIT(His) + empty entry(Myc) vector anti-Myc immunoprecipitation, (8) N209I ECSIT(His) + wild-type NDUFAF1/ACAD9(Myc) anti-His immunoprecipitation, (9) empty AC-His vector + wild-type NDUFAF1/ACAD9(Myc) anti-His immunoprecipitation, (10) N209I ECSIT(His) + wild-type NDUFAF1/ACAD9(Myc) anti-Myc immunoprecipitation, (11) N209I ECSIT + empty entry(Myc) vector anti-Myc immunoprecipitation (representative of *n* = 3 experimental repeats). Mean ± SEM, **P < 0.01.

Previous work has demonstrated that ECSIT has two main isoforms, a 50 kDa cytosolic isoform and a 45 kDa mitochondrial isoform that are formed following the cleavage of a 5 kDa mitochondrial-targeting sequence at the protein’s N terminus upon localization to the mitochondria.^8^ Western blot analysis demonstrated the presence of both isoforms in heart and brain tissue (*Figure 6A* and *B*), with a significant increase in both protein products in the heart tissue of mutants compared with wild type, without measurable differences in brain tissue. Interestingly, a third band, corresponding to a previously undescribed ∼16 kDa fragment, can be seen in the heart tissue of wild-type animals; this fragment is conspicuously absent from the heart tissue of *Ecsit^N209I/N209I^* animals as well as in brain, liver, and kidney (see Supplementary material online, *Figures S10* and *S11*) from both genotypes.

As with mitochondrial proteins above, ECSIT protein levels were assessed in heart of 2-week-old animals to confirm causation with the phenotype observed (see Supplementary material online, *Figure S7B*). These results demonstrate that the 16 kDa fragment discussed is reduced at an early time point, before the onset of cardiac disease. However, the larger protein products are unchanged in their abundance.

To further investigate the role of ECSIT in Complex I assembly and the role of the N209I mutation in its function, the interaction with known binding partners was assessed by co-immunoprecipitation. Tagged ECSIT (His) and either ACAD9 (Myc) or NDUFAF1 (Myc) were co-transfected into HEK293T cells. Immunoprecipitation was performed using antibodies against the relevant tags and western blot against the various isoforms performed.

As expected, immunoprecipitation with either anti-His or anti-Myc antibody was able to pull down both the wild-type ECSIT and NDUFAF1 proteins in both their cytosolic and mitochondrial isoforms (*Figure 6C*). The mutant N209I protein was also co-immunoprecipitated with ACAD9 and NDUFAF1.

Similarly, immunoprecipitation with ECSIT and ACAD9 (*Figure 6D*) demonstrated that both proteins can be pulled down using both the anti-His and anti-Myc antibodies that correspond to the relevant tagged proteins. The N209I mutation does not seem to significantly affect this protein–protein interaction, as the same results are demonstrated in lanes containing only N209I mutant ECSIT protein.

### 3.7 Complex I assembly processes in various tissues

**Figure 7:**
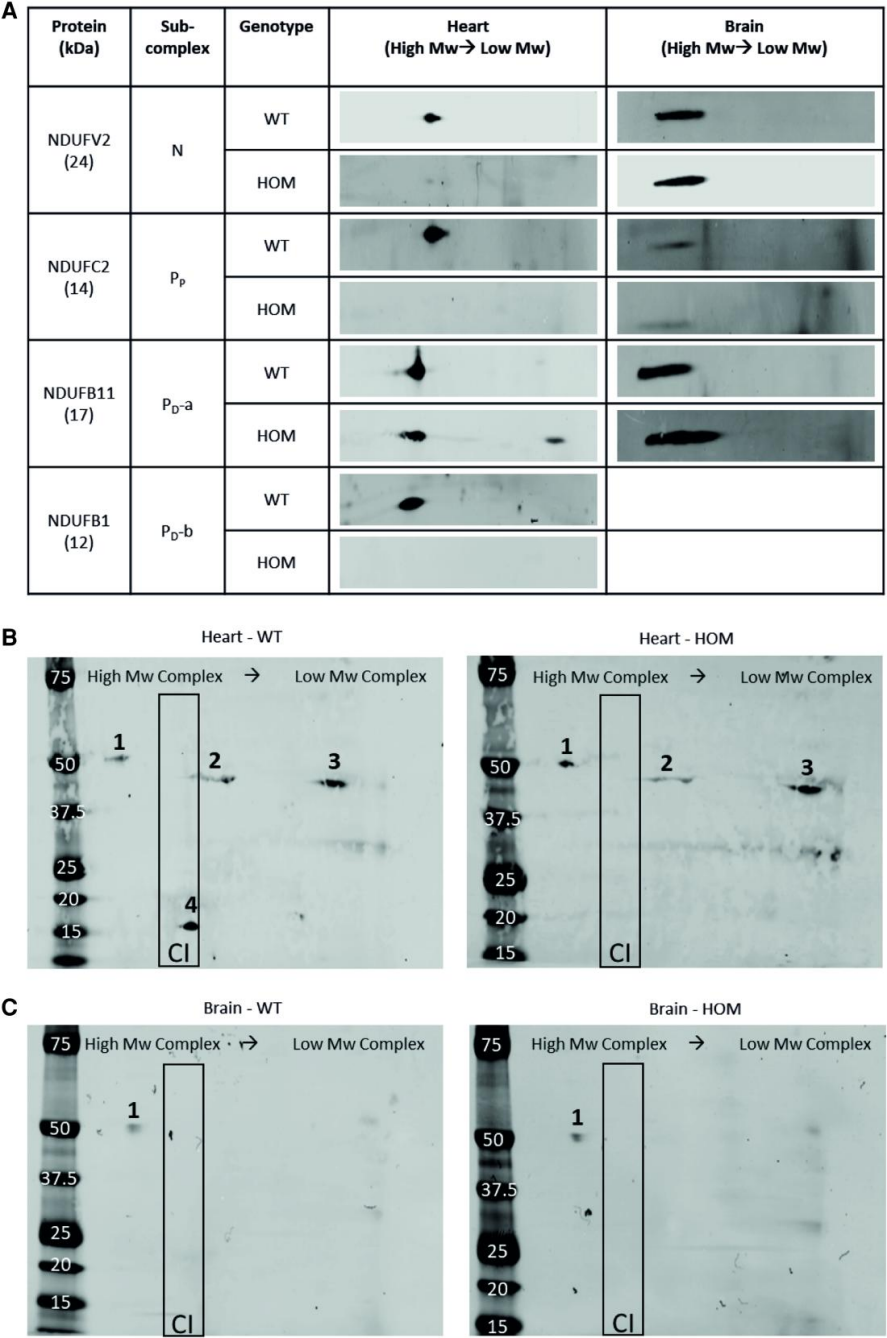
(*A*) Representative second-dimensional BN-PAGE blots comparing heart and brain complexes. In each panel, high-molecular weight complexes are on the left and low-weight complexes on the right. Differences seen in the patterning and assembly of Complex I subcomplexes in the heart were not seen in the same subcomplexes in the brain. (*B* and *C*) Second-dimensional BN-PAGE blots for ECSIT protein in heart and brain demonstrating 50 kDa (1) and 45 kDa (2 and 3) protein fractions as well as the newly identified 16 kDa fragment (4). Blots in brain mitochondria show an absence of both the 45 and 16 kDa protein fractions. The outlined area is the expected position for intact Complex I enzyme–associated proteins (*n* = 4).

Finally, to investigate how the assembly process of Complex I is altered in different tissues given the differences seen in Complex I protein abundance and activity, 2D-BN-PAGE was performed in various tissues from 16-week-old animals. Proteins representing each of the different portions of Complex I were chosen and the patterning of each determined in wild-type and *Ecsit^N209I/N209I^* heart tissue and brain tissue (*Figure 7A*). NDUFV2 (N), NDUFC2 (P_P_), and NDUFB1 (P_D_-b) demonstrated a markedly altered abundance in the portion of the band corresponding to intact Complex I in mutant animals, while NDUFB11 (P_D_-a) showed an accumulation at a band corresponding to an assembly intermediate or isolated protein in *Ecsit^N209I/N209I^* heart tissue. There were no notable changes to proteins in the remaining subcomplexes. The proteins that demonstrated changes in heart tissue were then tested in brain tissue and, with the exception of NDUFB1, which was undetectable by western blot, demonstrated no significant alterations in abundance or patterning between wild-type and mutant tissue.

2D-BN-PAGE was also performed against ECSIT protein to determine how the N209I mutation affects the assembly process of Complex I. Wild-type ECSIT protein (*Figure 7B*) can be seen in four individual bands on the 2D-BN-PAGE. The first represents the 50 kDA cytosolic isoform and can be seen in a high Mw complex, larger even than fully assembled Complex I, which we hypothesize to be the import machinery associated with the outer mitochondrial membrane. The 45 kDa mitochondrial fragment can be observed in two bands which correspond to Complex I assembly intermediates but is not present in a band that would represent completed Complex I. Finally, the 16 kDa fragment we had previously identified was present at a size approximately equal to the completed, intact Complex I holoenzyme. The pattern of the 50 and 45 kDa fragments of ECSIT is repeated in the 2D-BN-PAGE of the *Ecsit^N209I/N209I^* heart mitochondria, suggesting that the production of and import into the mitochondria of the mutant ECSIT protein is intact. However, the lack of the 16 kDa fragment is striking, given the presence of intact Complex I in the blots of many of the Complex I proteins shown previously. The absence of this fragment in fully assembled Complex I suggests that the N209I mutation interferes with the production of this fragment during the assembly process.

2D-BN-PAGE in brain mitochondria demonstrates the presence of the 50 kDa fragment in a single band (*Figure 7C*), as seen in the heart mitochondria. As expected, we were unable to detect the 16 kDa fragment by western blot in brain lysate (see Supplementary material online, *Figure S10*) and in addition, the 45 kDa protein product was not detectable by 2D-BN-PAGE.

Taken together, these data suggest that ECSIT is an essential component of MCIA in heart tissue, while its role in the brain seems to be less essential as both the 45 and 16 kDa fragments were absent from the Complex I assembly process and in the complete holoenzyme. Furthermore, it suggests that ECSIT is not only involved in the assembly intermediates of Complex I in heart mitochondria, but a portion of the protein forms an integral part of completed holoenzyme.

## 4. Discussion

Through a phenotype-driven screen, we have identified a point mutation in the MCIA factor resulting in tissue-specific effects on Complex I assembly, primarily affecting cardiac tissue, and resulting in progressive HCM. Mitochondria from *Ecsit^N209I/N209I^* hearts exhibited structural abnormalities, with the presence of hyper condensed, disorganized cristae, as well as a small reduction in mitochondrial cross-sectional area of both interfibrillar and perinuclear mitochondria. Protein and mtDNA levels did not demonstrate a change in the overall abundance of mitochondria in heart tissue, although the level of PGC1α, the master regulator of mitochondrial biogenesis, was elevated in heart tissue (∼50%). This result may indicate that while there is an up-regulation of the pathway that drives mitochondrial biogenesis, the mutation of ECSIT either inhibits the actual process or results in the production of defective mitochondria, or mitochondrial subunits, that are eliminated by either mitophagy or the mitochondrial unfolded protein response.

Analysis of Complex I protein levels confirmed that there was a drastic reduction in Complex I levels in heart, whereas other tissues (brain, kidney, liver, and skeletal muscle) exhibited a smaller reduction. The extent of reduction varies considerably between tissues with the brain showing only a small reduction of around 30%, while the heart shows a >95% reduction in NDUFB8 protein levels. This reduction in Complex I protein level is not reflected across all proteins of Complex I, with a more robust reduction in membrane arm proteins than in matrix arm proteins.

Results also demonstrated that, while Complex I protein levels are reduced in several tissues, the activity of Complex I is only affected in cardiac tissue. Comparing heart and brain (two tissues commonly affected by Complex I deficiency) using in-gel activity and seahorse analysis of isolated mitochondria, revealed that Complex I activity and mitochondrial respiration were significantly reduced in mutant tissue. However, this was not true for Complex I or mitochondria isolated from brain tissue, which showed comparable levels in wild-type and *Ecsit^N209I/N209I^* mitochondria in both assays. Similarly, the *Ecsit^N209I/N209I^* mitochondria had little effect on Complex I assembly in muscle and other tissues. These data suggest that there are tissue-specific differences in the Complex I assembly process, and in particular the stage(s) controlled by ECSIT. However, the mutation of ECSIT protein had no impact on the interaction between ECSIT and other proteins of the MCIA complex such as NDUFAF1 and ACAD9, which also showed no changes in protein levels in any tissues tested.

The tissue-specific effects of the *Ecsit^N209I/N209I^* mutation explain why the main phenotype observed was HCM. Of interest was the identification of a previously undescribed ∼16 kDa fragment in wild-type heart tissue, which was absent from *Ecsit^N209I/N209I^* hearts. ECSIT exists as a cytosolic (50 kDa) and mitochondrial (45 kDa) form in mouse tissues and the levels of these two protein products were higher in *Ecsit^N209I/N209I^* animals compared with wild type. The reason for this accumulation is unclear but the absence of the smaller 16 kDa ECSIT protein in cardiac tissue suggests a cleavage of ECSIT in normal tissue to facilitate the assembly of Complex I. It is conceivable that this fragment is a portion of ECSIT produced by the cleavage of the 45 kDa ECSIT as part of the normal assembly process for Complex I. If this is the case, then it may be that the N209I mutation inhibits this cleavage, leading to an accumulation of the larger 45 and 50 kDa ECSIT isoforms. In brain tissue, we only observed the 50 kDa form of ECSIT in BN-PAGE gels. This may be because ECSIT is not transported into the mitochondria in the brain. This fits with our observations that ECSIT is not required for MCIA in brain tissue.

To assess Complex I assembly, first- and second-dimensional BN-PAGE were performed on mitochondria extracts from heart and brain of wild-type and *Ecsit^N209I/N209I^* animals. This revealed that some aspects of Complex I assembly were defective in cardiac tissue from *Ecsit^N209I/N209I^* animals, but not in brain tissue. These defects mainly relate to the assembly of the membrane arm of Complex I, although some loss of other subunits was also seen. Interestingly, none of the same defects were seen in mitochondria isolated from brain tissue of the same animals. This further supports the hypothesis that Complex I assembly is not a universal process but has tissue-specific intricacies.

Taken together, these data suggest that the N209I mutation of ECSIT has no effect on protein synthesis, as indicated by the presence of the 50 and 45 kDa bands, or on the interaction between ECSIT and the MCIA proteins NDUFAF1 and ACAD9. Our novel finding was the presence of a 16 kDa fragment detected by the anti-ECSIT mAb. This fragment was not detected in mutant cardiac tissue or in wild-type and mutant brain tissue. Complex I assembly and activity in brain tissue were unaffected and those tissues tested that did show Complex I protein level differences are less severely affected than heart tissue.

In wild-type animals, ECSIT is expressed as full-length 50 kDa protein in all tissues and according to demand is poly-ubiquitinated and targeted to mitochondria, where it is imported and the mitochondrial-targeting sequence cleaved, leaving a 45 kDa ECSIT protein. Once localized to the mitochondria, ECSIT forms part of the MCIA complex along with NDUFAF1 and ACAD9.^6^ To this point in the Complex I assembly, our data suggest that ECSIT functions normally. The MCIA complex is then involved in the assembly of the membrane arm of Complex I.^8–10^ From this point, the mutant ECSIT protein prevents the normal assembly of Complex I in cardiac tissue.

However, the assembly process appears to be tissue specific; the N209I mutation varies in its effect on Complex I assembly in different tissues and there are even differences in the ECSIT protein association with Complex I between brain and cardiac tissue in wild-type animals. The different effects of the N209I mutation on Complex I assembly between tissues, possibly because of tissue-specific requirements for ECSIT in this process. Indeed, Xu *et al.*^24^ demonstrate that a murine model expressing human ECSIT maintains most known functions of Ecsit (BMP pathway, pro-inflammatory, NF-κB activation); they do demonstrate a critical role for ECSIT in mitochondrial function and hypothesize a ‘threshold level’ of expression. Taken together with the data in our present study, we can further suggest that rather than a threshold level of Ecsit being required, this is instead a tissue-specific effect of Ecsit cleavage and integration into complete Complex I holoenzyme.

While the model described herein represents a unique ENU-induced mutation, it highlights the potential for greater understanding of existing mutations. Where isolated cardiomyopathy is observed in the absence of other phenotypes as the result of an identified mitochondrial mutation, our work suggests that this might be explained by tissue-specific interactions of the identified protein. This may be either in the Complex I assembly process or in the functional Complex I enzyme itself. Further work on the structure and assembly of Complex I in human tissues will reveal the details of how mitochondria differ between tissues and highlight potential targets for treatment.

In summary, we have identified a novel model of HCM resulting from a mutation in the MCIA protein, ECSIT. The mutation has no effect on the TLR pathway but appears to affect the cleavage of ECSIT during MCIA. We also provide evidence for tissue-specific requirements for ECSIT in Complex I assembly, which explains the severe cardiac phenotype in the absence of other phenotypes and provides the first evidence of a mechanism underlying a tissue-specific effect of mitochondrial dysfunction. This mutant line provides opportunities to investigate the mechanisms underlying not only HCM but also tissue-specific differences in Complex I assembly. Furthermore, these findings also have implications for the clinical diagnosis of cardiac disease and testing procedures for Complex I assembly deficiencies, as this is often carried out using skin fibroblasts,^25–27^ which may not reflect Complex I assembly in all tissues.

## Supplementary Material

cvad101_Supplementary_Data

## Data Availability

All data underlying the findings reported and discussed in this article are available within the article and its online supplementary material. Further data not discussed or reported here are available upon reasonable request to the corresponding author.

## Notes

Translational perspective Mitochondrial diseases often present as multi-system disorders with far-reaching implications for the health and well-being of patients. Diagnoses are often undertaken by characterization of mitochondrial function from skin or muscle biopsy, with the expectation that any effect on mitochondrial function will be recognizable in all cell types. However, this study demonstrates that mitochondrial function may differ between cell types with the involvement of tissue-specific proteins or isoforms; as such, current diagnostic techniques may miss diagnoses of a more specific mitochondrial dysfunction.

## References

[1] Guerrero-Castillo S , Baertling F, Kownatzki D, Wessels HJ, Arnold S, Brandt U, Nijtmans L. The assembly pathway of mitochondrial respiratory chain complex I. Cell Metab 2017;25:128–139. 27720676 10.1016/j.cmet.2016.09.002

[2] Mimaki M , Wang X, McKenzie M, Thorburn DR, Ryan MT. Understanding mitochondrial complex I assembly in health and disease. Biochim Biophys Acta 2012;1817:851–862. 21924235 10.1016/j.bbabio.2011.08.010

[3] Sanchez-Caballero L , Guerrero-Castillo S, Nijtmans L. Unraveling the complexity of mitochondrial complex I assembly: a dynamic process. Biochim Biophys Acta 2016;1857:980–990. 27040506 10.1016/j.bbabio.2016.03.031

[4] Kopp E , Medzhitov R, Carothers J, Xiao C, Douglas I, Janeway CA, Ghosh S. ECSIT is an evolutionarily conserved intermediate in the Toll/IL-1 signal transduction pathway. Genes Dev 1999;13:2059–2071. 10465784 10.1101/gad.13.16.2059 PMC316957

[5] Xiao C , Shim JH, Kluppel M, Zhang SS, Dong C, Flavell RA, Fu XY, Wrana JL, Hogan BL, Ghosh S. Ecsit is required for Bmp signaling and mesoderm formation during mouse embryogenesis. Genes Dev 2003;17:2933–2949. 14633973 10.1101/gad.1145603 PMC289152

[6] Giachin G , Bouverot R, Acajjaoui S, Pantalone S, Soler-Lopez M. Dynamics of human mitochondrial complex I assembly: implications for neurodegenerative diseases. Front Mol Biosci 2016;3:43. 27597947 10.3389/fmolb.2016.00043 PMC4992684

[7] Kelley LA , Mezulis S, Yates CM, Wass MN, Sternberg MJ. The Phyre2 web portal for protein modeling, prediction and analysis. Nat Protoc 2015;10:845–858. 25950237 10.1038/nprot.2015.053 PMC5298202

[8] Vogel RO , Janssen RJ, van den Brand MA, Dieteren CE, Verkaart S, Koopman WJ, Willems PH, Pluk W, van den Heuvel LP, Smeitink JA, Nijtmans LG. Cytosolic signaling protein Ecsit also localizes to mitochondria where it interacts with chaperone NDUFAF1 and functions in complex I assembly. Genes Dev 2007;21:615–624. 17344420 10.1101/gad.408407 PMC1820902

[9] Nouws J , Nijtmans L, Houten SM, van den Brand M, Huynen M, Venselaar H, Hoefs S, Gloerich J, Kronick J, Hutchin T, Willems P, Rodenburg R, Wanders R, van den Heuvel L, Smeitink J, Vogel RO. Acyl-CoA dehydrogenase 9 is required for the biogenesis of oxidative phosphorylation complex I. Cell Metab 2010;12:283–294. 20816094 10.1016/j.cmet.2010.08.002

[10] Heide H , Bleier L, Steger M, Ackermann J, Drose S, Schwamb B, Zornig M, Reichert AS, Koch I, Wittig I, Brandt U. Complexome profiling identifies TMEM126B as a component of the mitochondrial complex I assembly complex. Cell Metab 2012;16:538–549. 22982022 10.1016/j.cmet.2012.08.009

[11] Rahman S , Blok RB, Dahl HH, Danks DM, Kirby DM, Chow CW, Christodoulou J, Thorburn DR. Leigh syndrome: clinical features and biochemical and DNA abnormalities. Ann Neurol 1996;39:343–351. 8602753 10.1002/ana.410390311

[12] Loeffen J , Smeitink J, Triepels R, Smeets R, Schuelke M, Sengers R, Trijbels F, Hamel B, Mullaart R, van den Heuvel L. The first nuclear-encoded complex I mutation in a patient with Leigh syndrome. Am J Hum Genet 1998;63:1598–1608. 9837812 10.1086/302154 PMC1377631

[13] Pavlakis SG , Phillips PC, DiMauro S, De Vivo DC, Rowland LP. Mitochondrial myopathy, encephalopathy, lactic acidosis, and strokelike episodes: a distinctive clinical syndrome. Ann Neurol 1984;16:481–488. 6093682 10.1002/ana.410160409

[14] Sproule DM , Kaufmann P. Mitochondrial encephalopathy, lactic acidosis, and strokelike episodes: basic concepts, clinical phenotype, and therapeutic management of MELAS syndrome. Ann N Y Acad Sci 2008;1142:133–158. 18990125 10.1196/annals.1444.011

[15] Loeffen J , Elpeleg O, Smeitink J, Smeets R, Stöckler-Ipsiroglu S, Mandel H, Sengers R, Trijbels F, van den Heuvel L. Mutations in the complex I NDUFS2 gene of patients with cardiomyopathy and encephalomyopathy. Ann Neurol 2001;49:195–201. 11220739 10.1002/1531-8249(20010201)49:2<195::aid-ana39>3.0.co;2-m

[16] Benit P , Beugnot R, Chretien D, Giurgea I, De Lonlay-Debeney P, Issartel JP, Corral-Debrinski M, Kerscher S, Rustin P, Rotig A, Munnich A. Mutant NDUFV2 subunit of mitochondrial complex I causes early onset hypertrophic cardiomyopathy and encephalopathy. Hum Mutat 2003;21:582–586. 12754703 10.1002/humu.10225

[17] Fassone E , Taanman JW, Hargreaves IP, Sebire NJ, Cleary MA, Burch M, Rahman S. Mutations in the mitochondrial complex I assembly factor NDUFAF1 cause fatal infantile hypertrophic cardiomyopathy. J Med Genet 2011;48:691–697. 21931170 10.1136/jmedgenet-2011-100340

[18] Sanchez-Caballero L , Ruzzenente B, Bianchi L, Assouline Z, Barcia G, Metodiev MD, Rio M, Funalot B, van den Brand MA, Guerrero-Castillo S, Molenaar JP, Koolen D, Brandt U, Rodenburg RJ, Nijtmans LG, Rotig A. Mutations in complex I assembly factor TMEM126B result in muscle weakness and isolated complex I deficiency. Am J Hum Genet 2016;99:208–216. 27374773 10.1016/j.ajhg.2016.05.022 PMC5005453

[19] Alston CL , Compton AG, Formosa LE, Strecker V, Olahova M, Haack TB, Smet J, Stouffs K, Diakumis P, Ciara E, Cassiman D, Romain N, Yarham JW, He L, De Paepe B, Vanlander AV, Seneca S, Feichtinger RG, Ploski R, Rokicki D, Pronicka E, Haller RG, Van Hove JL, Bahlo M, Mayr JA, Van Coster R, Prokisch H, Wittig I, Ryan MT, Thorburn DR, Taylor RW. Biallelic mutations in TMEM126B cause severe complex I deficiency with a variable clinical phenotype. Am J Hum Genet 2016;99:217–227. 27374774 10.1016/j.ajhg.2016.05.021 PMC5005451

[20] Ghezzi D , Zeviani M. Human diseases associated with defects in assembly of OXPHOS complexes. Essays Biochem 2018;62:271–286. 30030362 10.1042/EBC20170099 PMC6056716

[21] Potter PK , Bowl MR, Jeyarajan P, Wisby L, Blease A, Goldsworthy ME, Simon MM, Greenaway S, Michel V, Barnard A, Aguilar C, Agnew T, Banks G, Blake A, Chessum L, Dorning J, Falcone S, Goosey L, Harris S, Haynes A, Heise I, Hillier R, Hough T, Hoslin A, Hutchison M, King R, Kumar S, Lad HV, Law G, MacLaren RE, Morse S, Nicol T, Parker A, Pickford K, Sethi S, Starbuck B, Stelma F, Cheeseman M, Cross SH, Foster RG, Jackson IJ, Peirson SN, Thakker RV, Vincent T, Scudamore C, Wells S, El-Amraoui A, Petit C, Acevedo-Arozena A, Nolan PM, Cox R, Mallon AM, Brown SD. Novel gene function revealed by mouse mutagenesis screens for models of age-related disease. Nat Commun 2016;7:12444. 27534441 10.1038/ncomms12444 PMC4992138

[22] Vikhe PP , Tateossian H, Bharj G, Brown SDM, Hood DW. Mutation in Fbxo11 leads to altered immune cell content in Jeff mouse model of otitis media. Front Genet 2020;11:50. 32117459 10.3389/fgene.2020.00050 PMC7026503

[23] Wi SM , Moon G, Kim J, Kim ST, Shim JH, Chun E, Lee KY. TAK1-ECSIT-TRAF6 complex plays a key role in the TLR4 signal to activate NF-kappaB. J Biol Chem 2014;289:35205–35214. 25371197 10.1074/jbc.M114.597187 PMC4271209

[24] Xu L , Humphries F, Delagic N, Wang B, Holland A, Edgar KS, Hombrebueno JR, Stolz DB, Oleszycka E, Rodgers AM, Glezeva N, McDonald K, Watson CJ, Ledwidge MT, Ingram RJ, Grieve DJ, Moynagh PN. ECSIT is a critical limiting factor for cardiac function. JCI Insight 2021;6:e142801. 10.1172/jci.insight.142801 PMC8262467 34032637

[25] Baertling F , Sanchez-Caballero L, van den Brand MAM, Fung CW, Chan SH, Wong VC, Hellebrekers DME, de Coo IFM, Smeitink JAM, Rodenburg RJT, Nijtmans LGJ. NDUFA9 point mutations cause a variable mitochondrial complex I assembly defect. Clin Genet 2017;93:111–118. 28671271 10.1111/cge.13089

[26] Koopman WJ , Verkaart S, van Emst-de Vries SE, Grefte S, Smeitink JA, Nijtmans LG, Willems PH. Mitigation of NADH: ubiquinone oxidoreductase deficiency by chronic Trolox treatment. Biochim Biophys Acta 2008;1777:853–859. 18435906 10.1016/j.bbabio.2008.03.028

[27] Verkaart S , Koopman WJ, Cheek J, van Emst-de Vries SE, van den Heuvel LW, Smeitink JA, Willems PH. Mitochondrial and cytosolic thiol redox state are not detectably altered in isolated human NADH:ubiquinone oxidoreductase deficiency. Biochim Biophys Acta 2007;1772:1041–1051. 17600689 10.1016/j.bbadis.2007.05.004

