# Supplementary material for: Tissue-specific differences in the assembly of mitochondrial Complex I are revealed by a novel ENU mutation in ECSIT
Source: Cardiovasc Res. 2023 Jul 3;119(12):2213–29. doi: 10.1093/cvr/cvad101 (PMC10578914; doi:10.1093/cvr/cvad101)
Supplement: cvad101_Supplementary_Data [file cvad101_supplementary_data.docx]

**
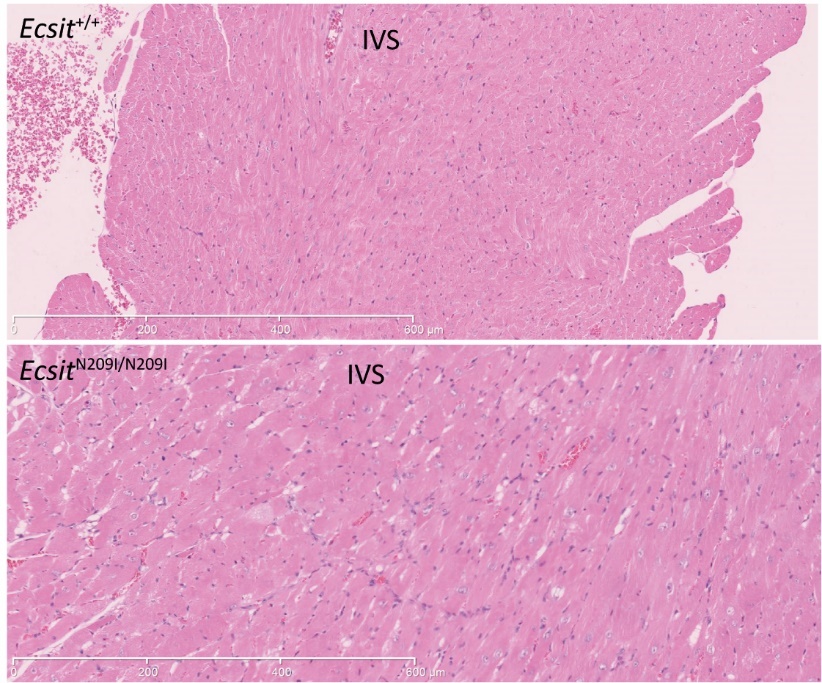
**

**Supplemental figure 1:** Haemotoxylin and Eosin stained histological section of wild-type and *Ecsit^N209I/N209I^* cardiac tissue demonstrating enlargement, disorganisation and vacuolation of the cardiomyocytes in the intraventricular septum (IVS) of *Ecsit^N209/N209I^* hearts in comparison to wild type. Scale bar = 600µm


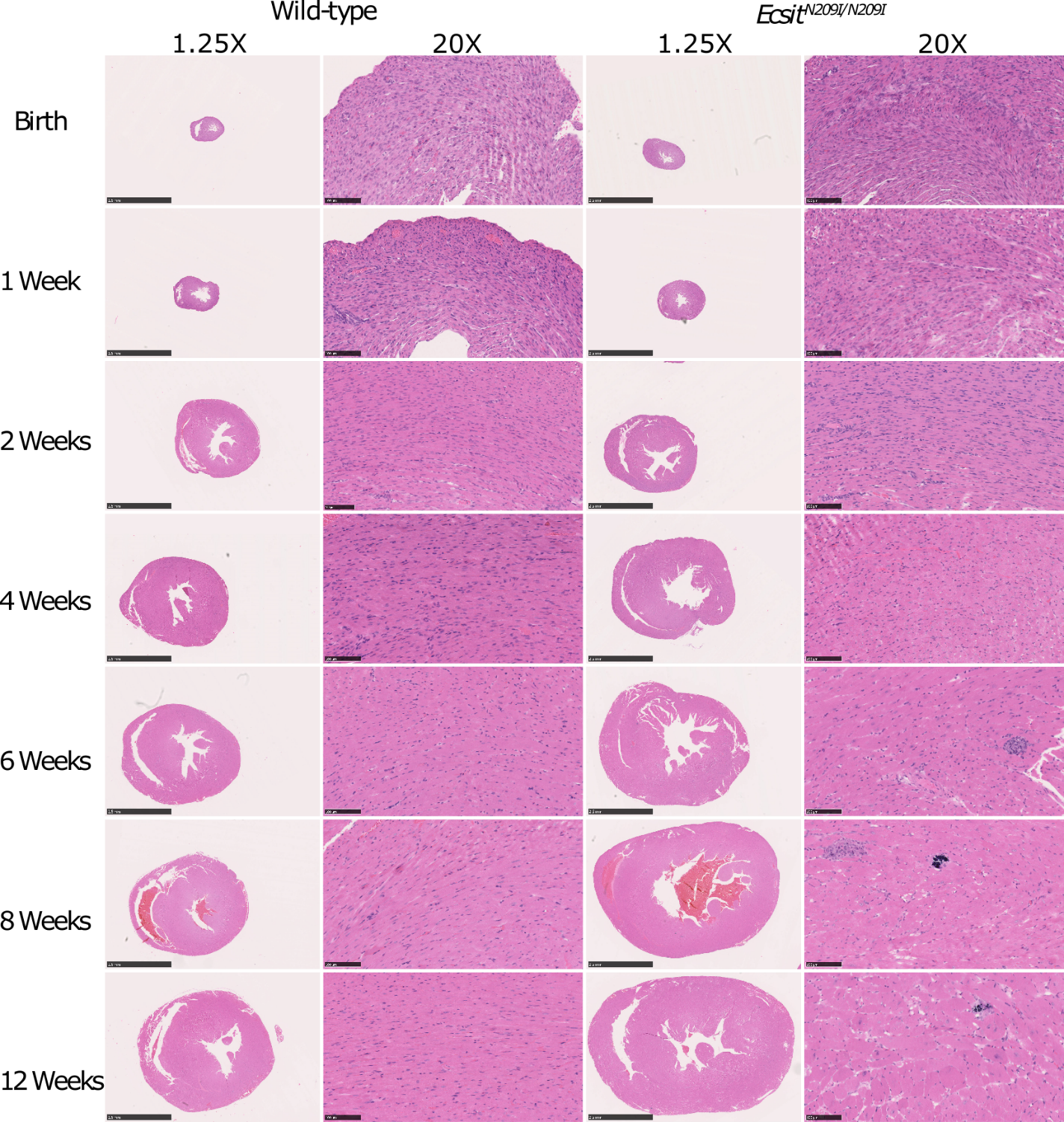


**Supplemental figure 2:** Haemotoxylin and Eosin stained histological sections of wild-type and Ecsit^N209I/N209I^ cardiac tissue at time points from birth to 12 weeks of age demonstrating progression of hypertrophy at 1.25x and 20x magnification. Representative of n=3 at each time point. Scale bar 2.5mm and 100µm.

**
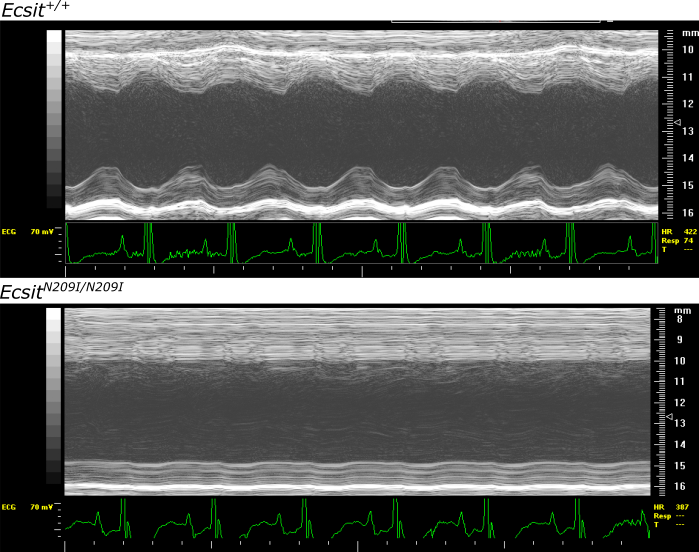
**

**Supplemental figure 3:** Representative echocardiographic images in M-Mode of *Ecsit^+/+^* and *Ecsit^N209I/N209I^* animals demonstrating reduced contractility of the heart muscle

**
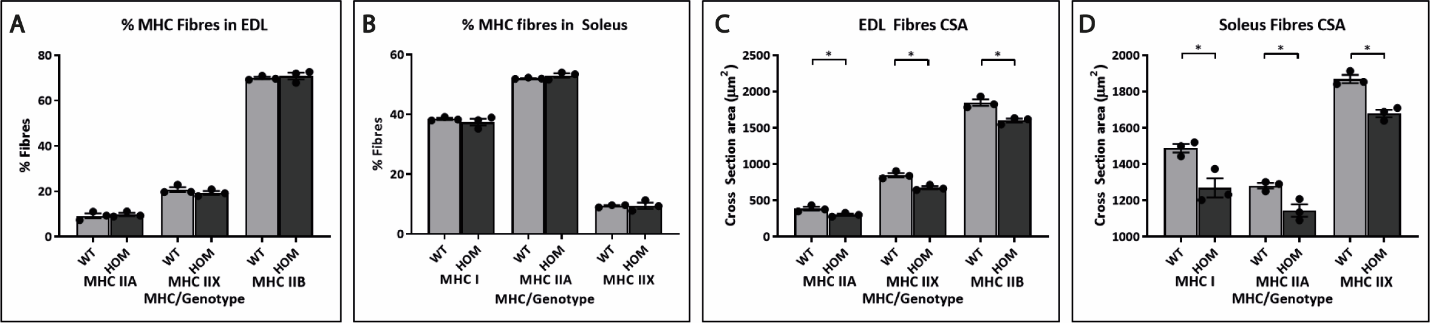
**

**Supplemental figure 4:** (A) Proportion of myosin heavy chain fibre types in the extensor digitorum longus (EDL) of wild-type and *Ecsit^N209I/N209I^* animals. (n=3, unpaired t-test) (B) Proportion of myosin heavy chain fibre types in the soleus of wild-type and *Ecsit^N209I/N209I^* animals. (n=3, unpaired t-test) (C) Cross sectional area (CSA) of myosin heavy chain fibre types in the EDL of wild-type and *Ecsit^N209I/N209I^* animals (n=3, unpaired t-test). (D) Cross sectional area (CSA) of myosin heavy chain fibre types in the soleus of wild-type and *Ecsit^N209I/N209I^* animals (n=3, unpaired t-test). Mean ± SEM, *p<0.05.

**
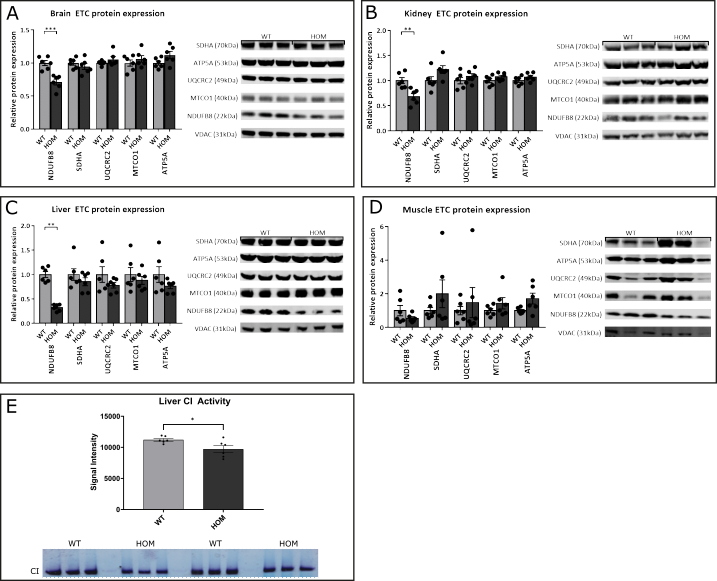
**

**Supplemental figure 5:** Abundance and representative blots of electron transport chain proteins (CI – NDUFB8, CII – SDHA, CIII – UQCRC2, CIV – MTCO1, CV – ATP5A) in brain (A), kidney (B), liver (C) and skeletal muscle (D) of wild-type and *Ecsit^N209I/N209I^* animals (n=6, unpaired t-test). (E) In gel activity assay of complex I in mitochondria from liver. Results show a slight reduction in activity of complex I in liver. Mean ± SEM, **p< 0.01, ***p<0.001.

**
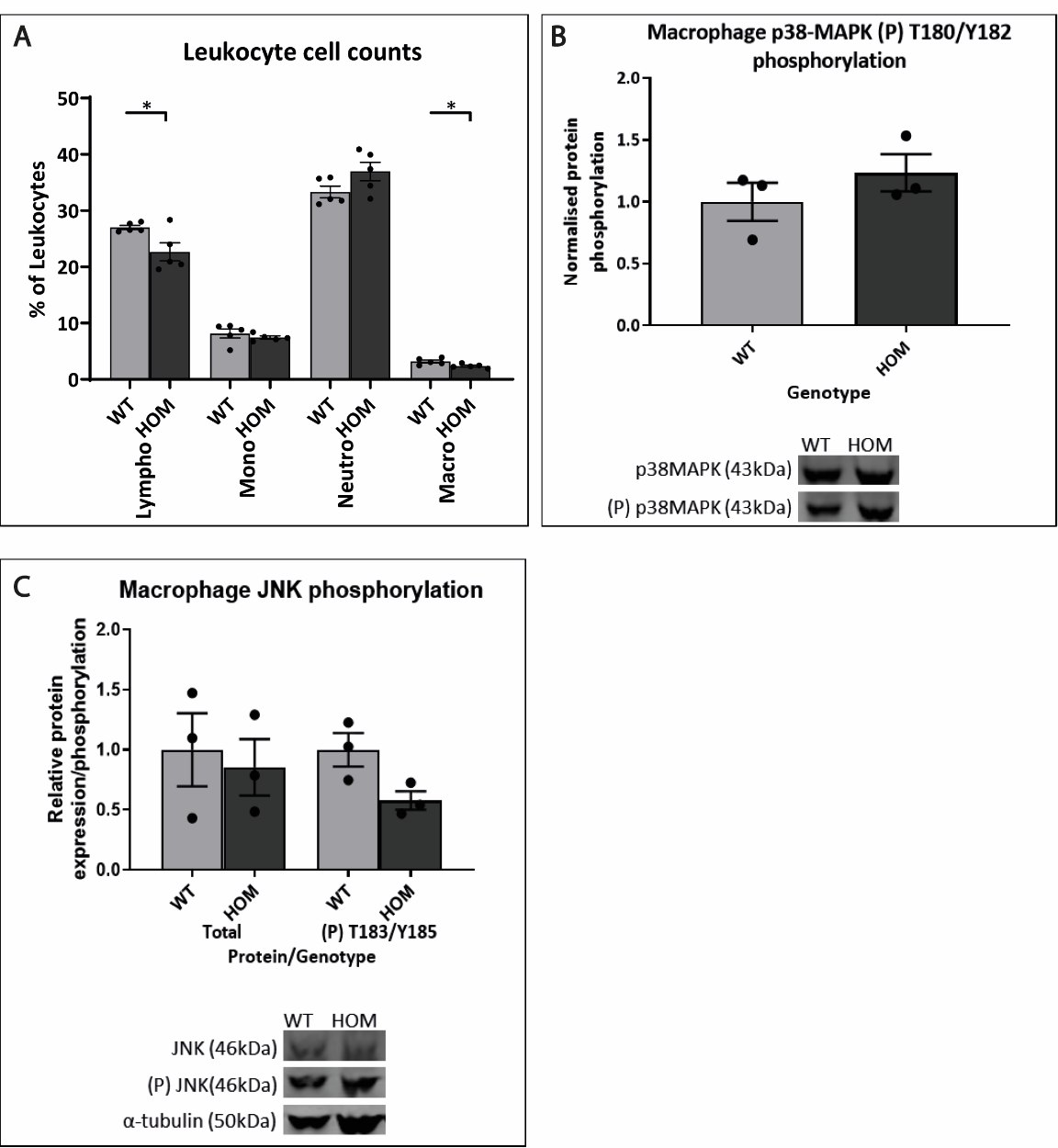
**

**Supplemental figure 6:** (A) Proportions of leukocyte cell types in wild-type and *Ecsit^N209I/N209I^* blood samples from 12-week-old animals (n=5, unpaired t-test). (B) Quantification and representative blot of p38-MAPK and phospho-p38-MAPK (T180/Y182) protein in stimulated (+LPS) bone marrow derived macrophages from wild-type and *Ecsit^N209I/N209I^* animals (n=3, unpaired t-test). (C) Quantification and representative blot of JNK and phospho-JNK (T183/Y185) protein in stimulated (+LPS) bone marrow derived macrophages from wild-type and *Ecsit^N209I/N209I^* animals (n=3, unpaired t-test). Mean ± SEM, *p<0.05.


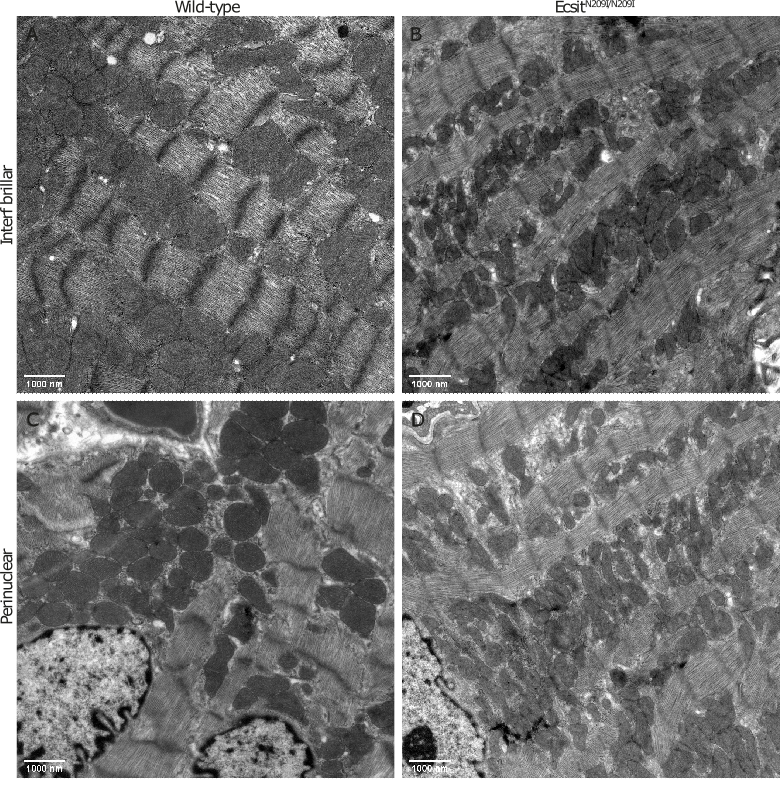


**Supplemental figure 7**: Low magnification transmission electron micrographs of Interfibrillar (A and B) and Perinuclear (C and D) mitochondria in heart tissue of wild-type and *Ecsit^N209I/N209I^* animals.


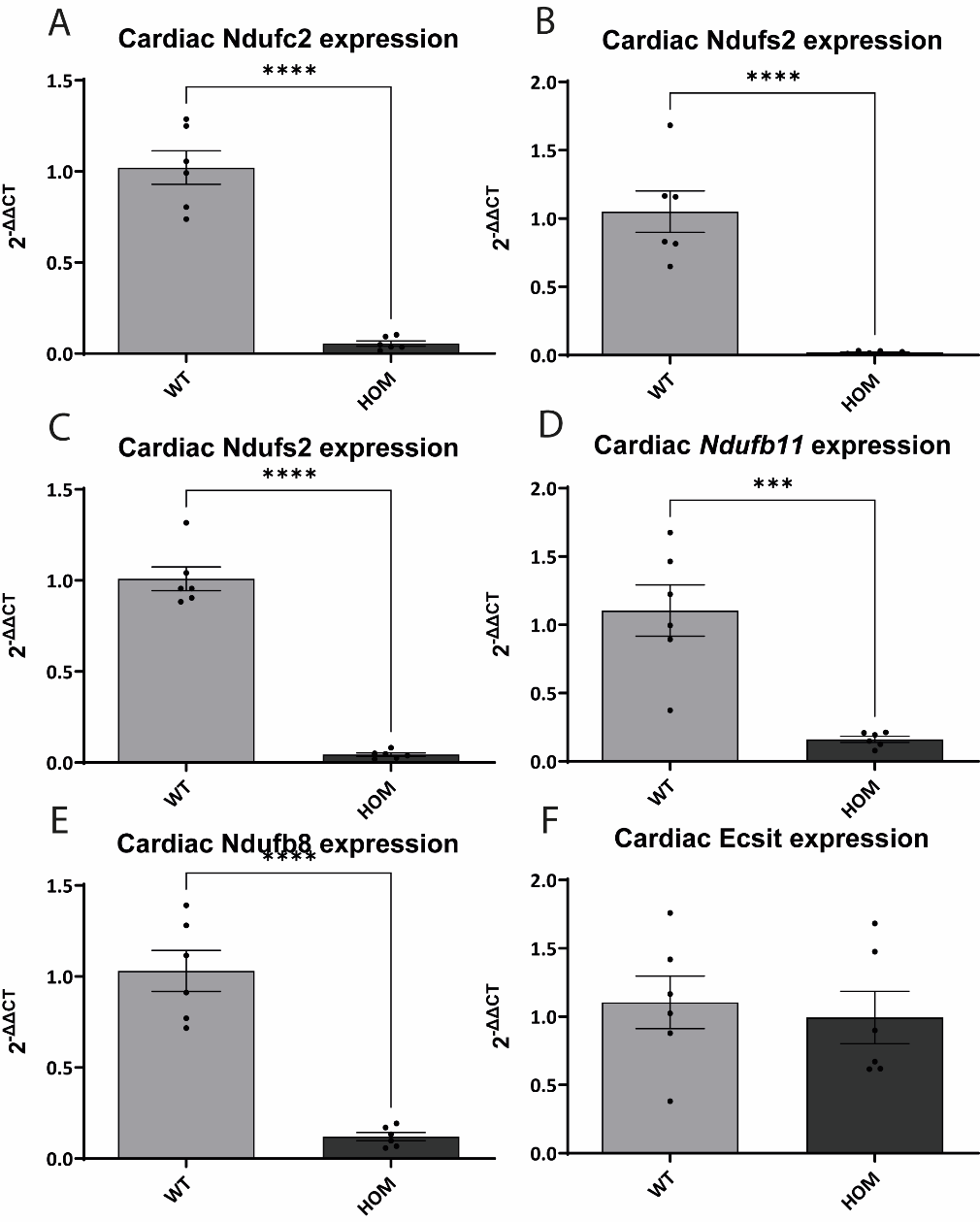


**Supplemental figure 8**: (A-E) Gene expression of mitochondrial complex I proteins in cardiac tissue corresponding to various subunits of complex I (N – NDUFV2, Q – NDUFS2, P­­_p_ – NDUFC2, P_D_ – NDUFB11, NDUFB8). Results demonstrate a marked loss of gene expression of all genes in agreement with protein level expression which was also significantly reduced (n=6, unpaired t-test). (F) Gene expression of Ecsit in cardiac tissue demonstrating that despite changes in protein levels of the various ECSIT fragments described, there is no alteration to Ecsit gene expression (n=6, unpaired t-test). (G) gene expression of PGC1a (n=6, unpaired t-test). Mean ± SEM, ***p< 0.001, ****p<0.0001


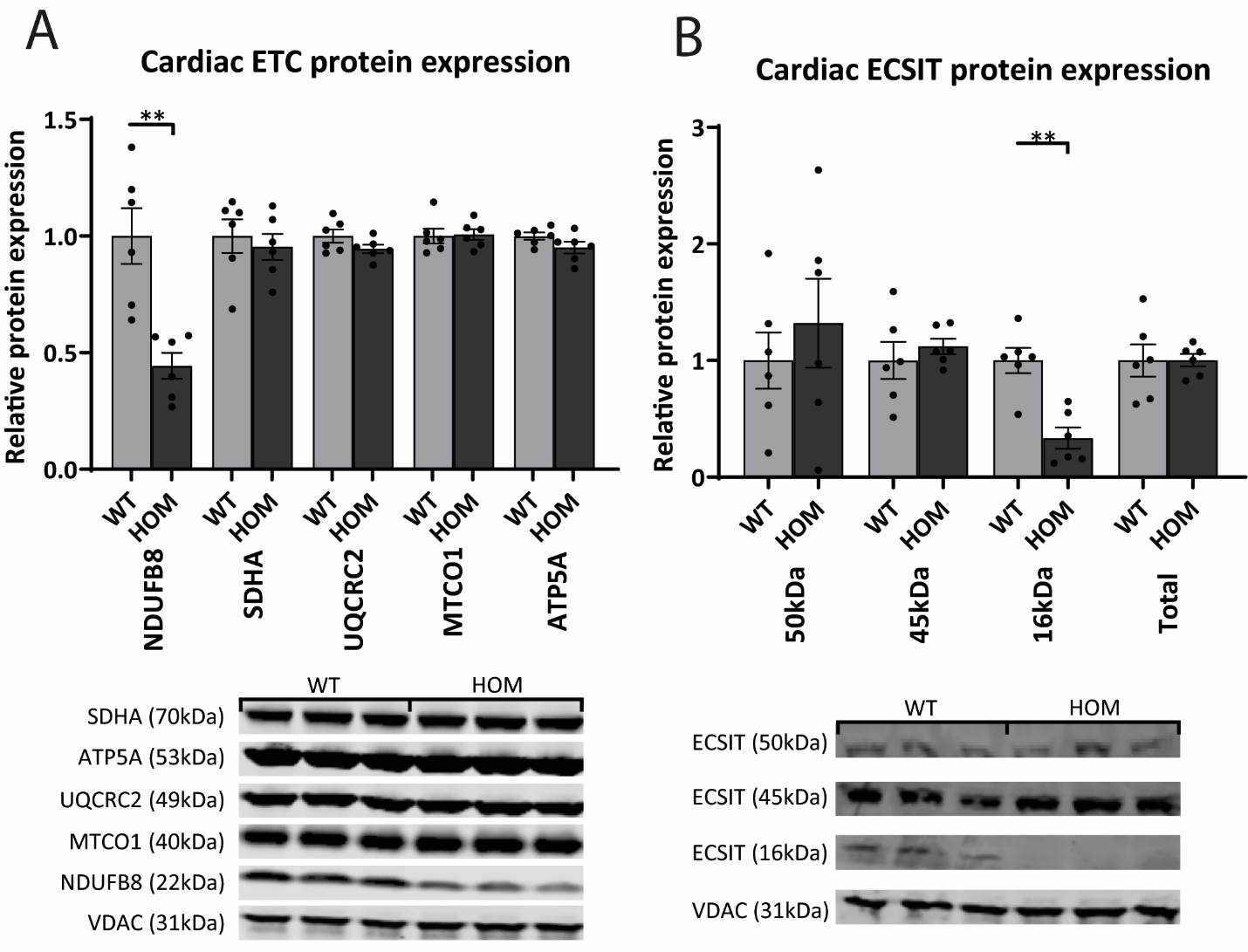


**Supplemental figure 9**: (A) Abundance and representative blots of electron transport chain proteins in cardiac tissue from 2-week-old wildtype and *Ecsit^N209I/N209I^* animals (CI – NDUFB8, CII – SDHA, CIII – UQCRC2, CIV – MTCO1, CV – ATP5A) demonstrating a loss of complex I protein abundance at 2 weeks of age, before the onset of cardiac phenotypes (n=6, unpaired t-test). (B) Quantification and representative blots of various ECSIT protein fragments in in cardiac tissue from 2-week-old wildtype and *Ecsit^N209I/N209I^* animals demonstrating a loss of abundance in the 16kDa fragment described whilst the remaining fragments are maintained at wildtype levels (n=6, unpaired t-test). Mean ± SEM, **p< 0.01

**
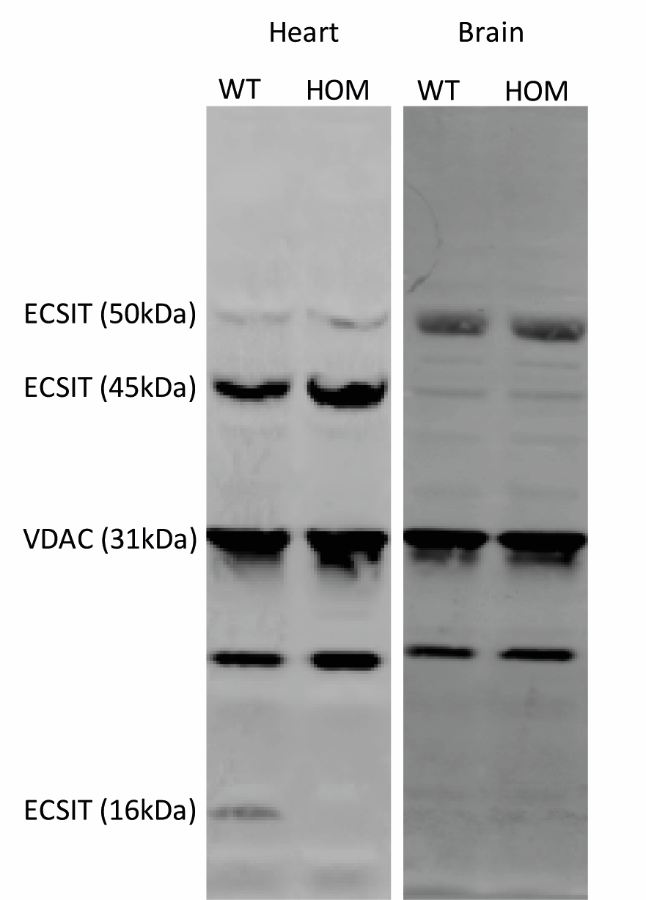
**

**Supplemental figure 10:** Representative blots of ECSIT protein in wild-type and *Ecsit^N209I/N209I^* hearts and brains demonstrating different abundances of various fragments of the ECSIT protein (representative images of n=3 experimental repeats).

**
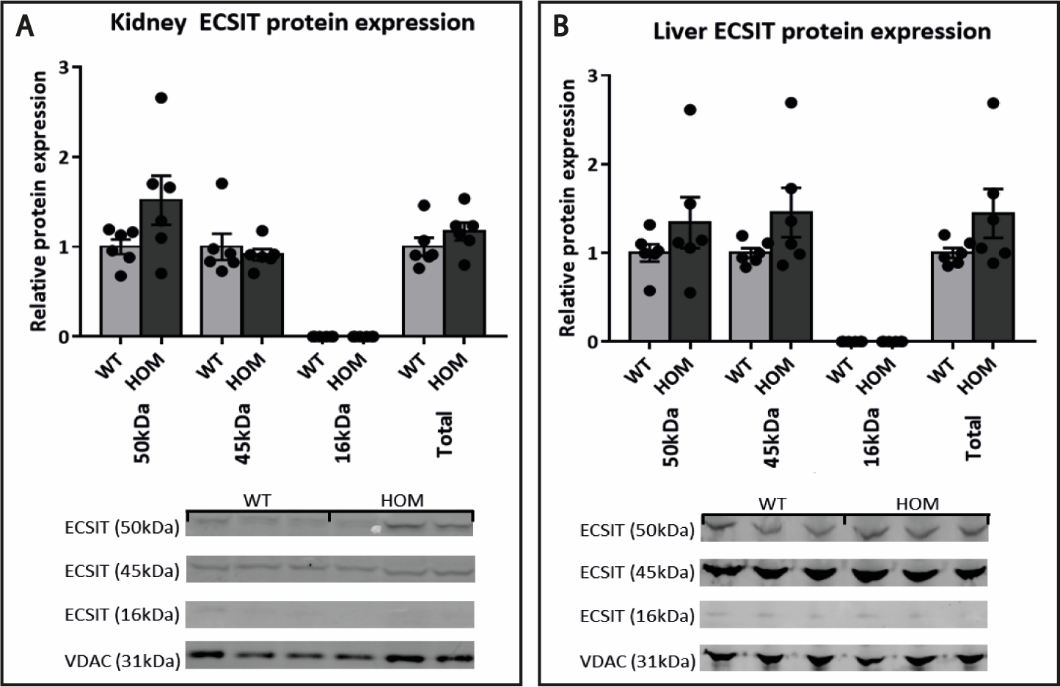
**

**Supplemental figure 11:** Quantification and representative blots of various ECSIT protein fragments in kidney (A) and liver (B) of wild-type and *Ecsit^N209I/N209I^* animals (n=6, unpaired t-test). No significant differences.

**Additional supplements – Complete Immunoblot images**

In all examples the section corresponding to the figure in question is highlighted in yellow.

Corresponding to Figure 3A


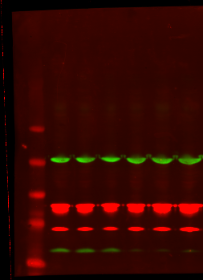


Atp5a/Ndufb8/Vdac


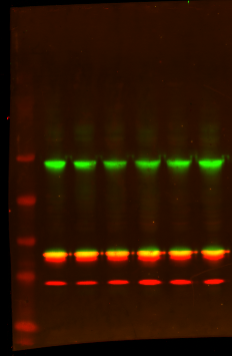


Mtco1/Sdha/Vdac


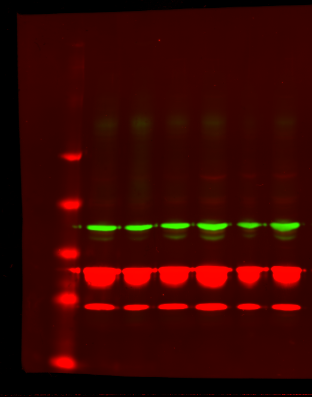


Uqcrc2/Vdac

Corresponding to Figure 4I


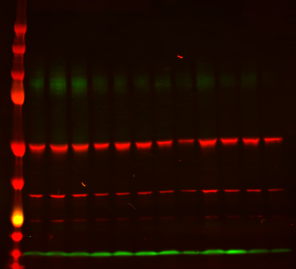


CoxIV/a-tubulin

Corresponding to Figure 4J


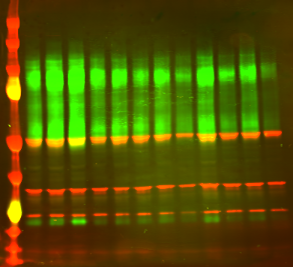


Tomm20/A-tubulin

Corresponding to Figure 4L


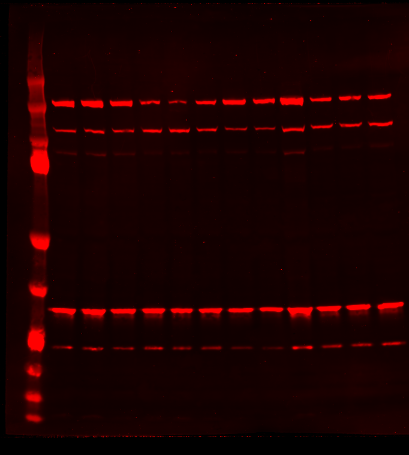


Pgc1a/Vdac

Corresponding to Figure 4N


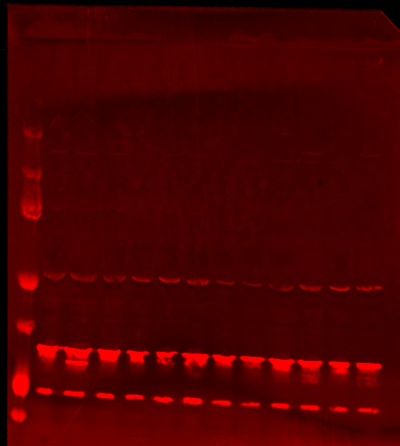


Pink1/Vdac

Corresponding to Figure 4O


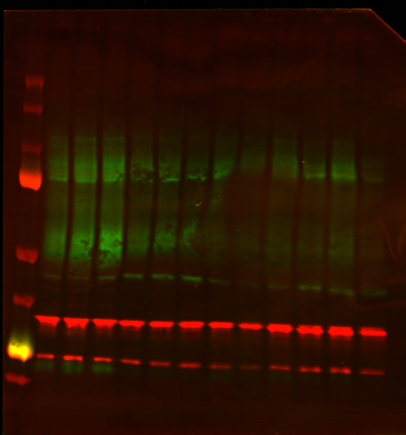


Mfn2/Vdac

Corresponding to Figure 4P


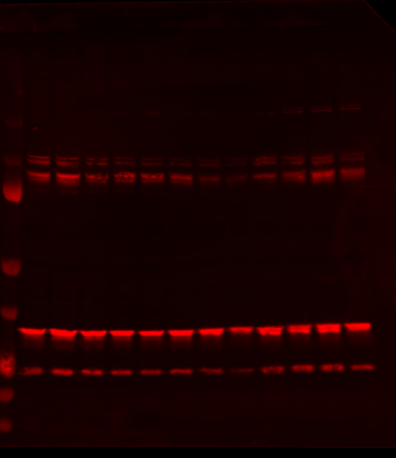


Opa1/Vdac

Corresponding to Figure 4Q


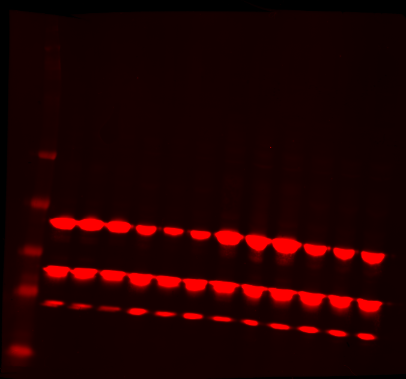


Ndufs2/Vdac


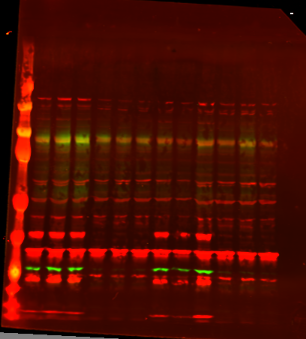


Complex I cocktail (NDUFA10, NDUFS3, NDUFV2, NDUFA8, NDUFC2, VDAC)


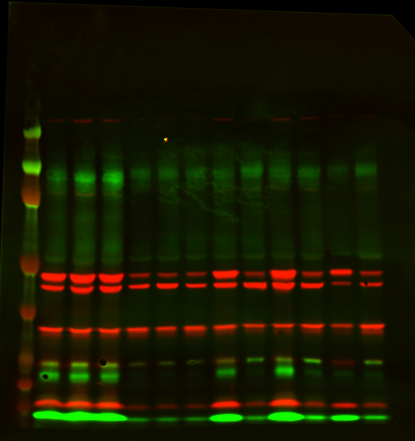


Complex I cocktail (NDUFS2, MT-ND1, NDUFS8, NDUFB11, NDUFB3, VDAC)

Corresponding to figure 5A


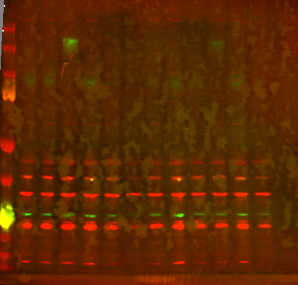


Complex I cocktail (Ndufv2, Ndufc2, Ndufa10, Ndufa8, Ndufs3, Vdac)


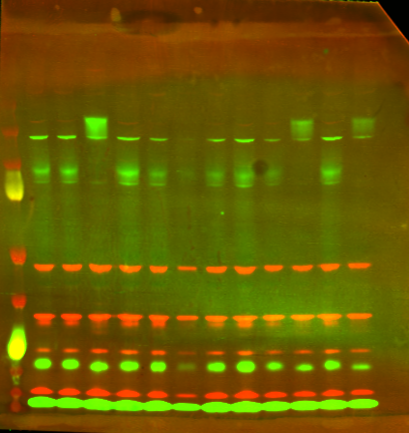


Complex I cocktail (NDUFB11, Mt-ND1, NDUFS2, NDUFB3, NDUFS8, Vdac)

Corresponding to Figure 6A


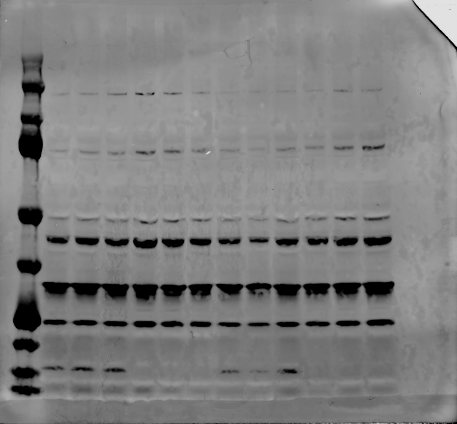


Ecsit, Vdac

Corresponding to Figure 6B


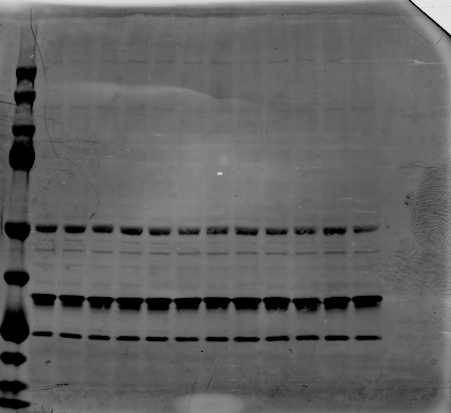


ECSIT, VDAC

Corresponding to Figure 7A

2 dimensional blots represented in Figure 7A have been adjusted in size to fit the figure and ensure the full width of the blot is demonstrated. As such, some bands in Figure 7 may appear horizontally compressed in comparison to the full blot demonstrated below.


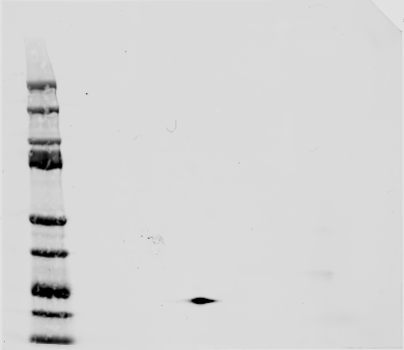


NDUFV2 – WT Heart


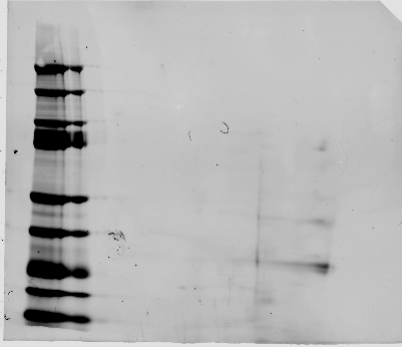


NDUFV2 – Hom Heart


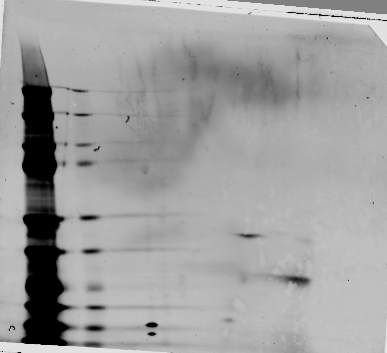


NDUFC2/ECSIT – WT Heart


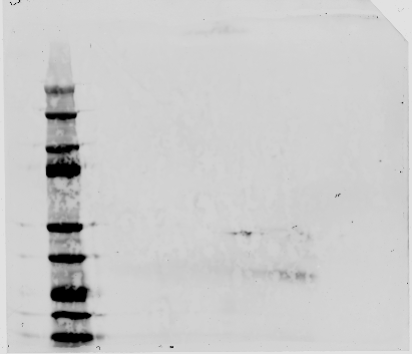


NDUFC2/ECSIT – HOM Heart


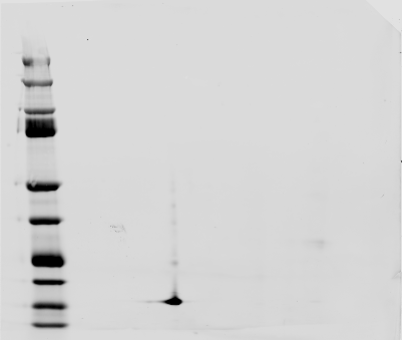


NDUFB11 – WT Heart


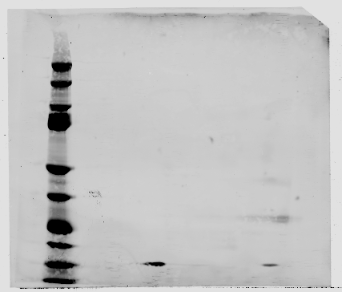


NDUFB11 – HOM Heart


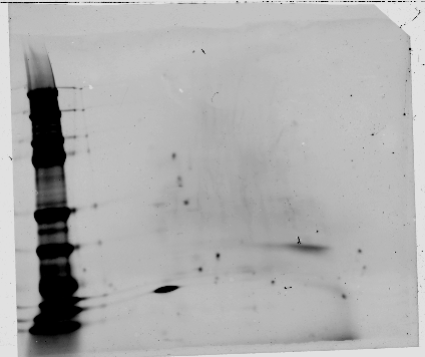


NDUFB1 – WT Heart


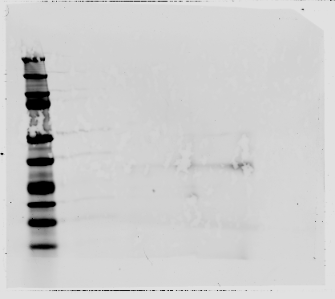


NDUFB1 – HOM Heart


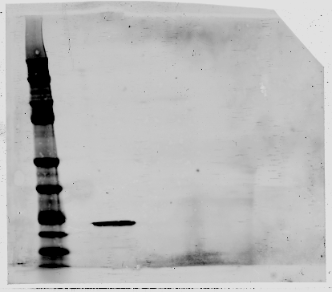


NDUFV2 – WT Brain


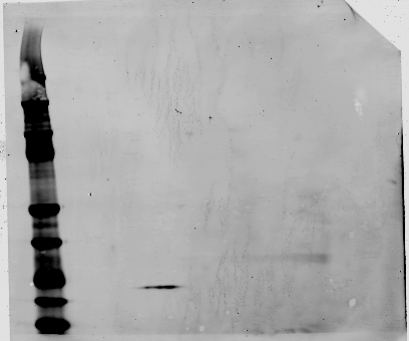


NDUFV2 – HOM Brain


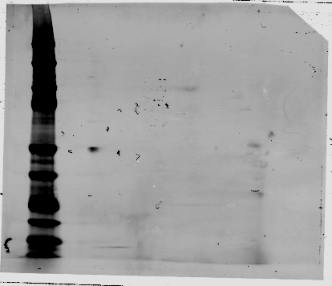


NDUFC2/ECSIT – WT Brain


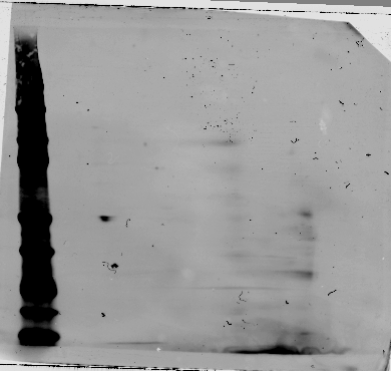


NDUFC2/ECSIT – HOM Brain

**
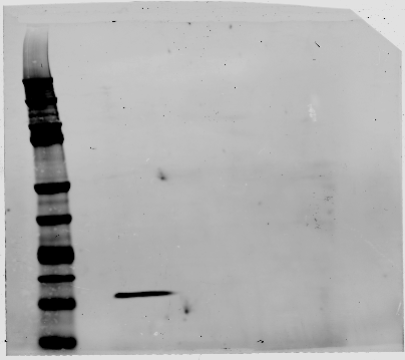
**

NDUFB1 – WT Brain


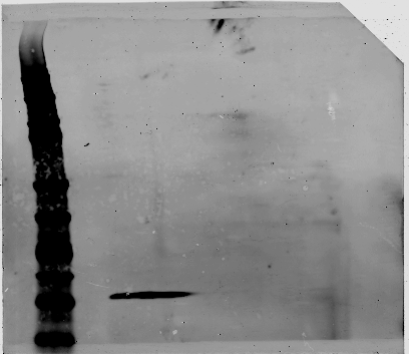


NDUFB1 – HOM Brain

Corresponding to Supplemental figure 5A


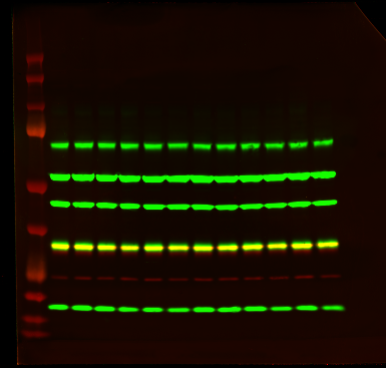


ETC protein cocktail (SDHA, ATP5A, UQCRC2, MTCO1, VDAC, NDUFB8) – Brain


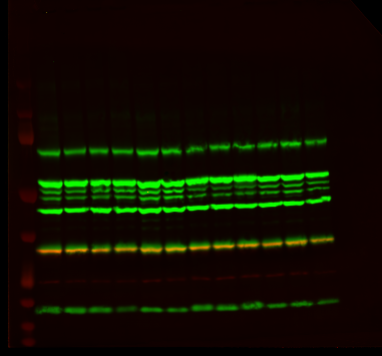


ETC protein cocktail (SDHA, ATP5A, UQCRC2, MTCO1, VDAC, NDUFB8) – Kidney


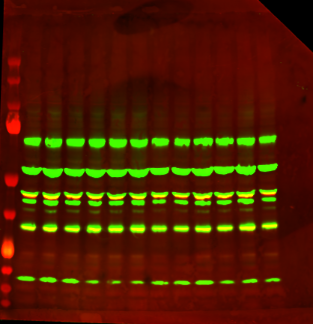


ETC protein cocktail (SDHA, ATP5A, UQCRC2, MTCO1, VDAC, NDUFB8) – Liver


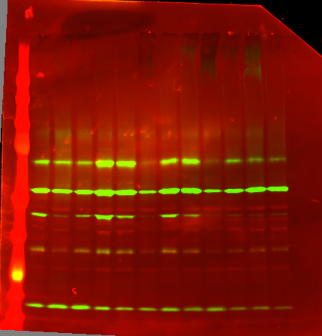


ETC protein cocktail (SDHA, ATP5A, UQCRC2, MTCO1, VDAC, NDUFB8) – Muscle

Corresponding to Supplemental Figure 6B


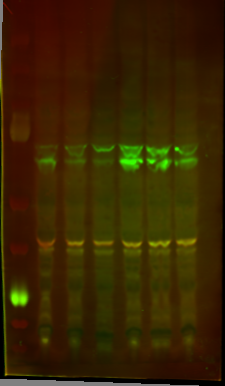


Total P38MapK (Red)/(P) P38 MapK (Green)


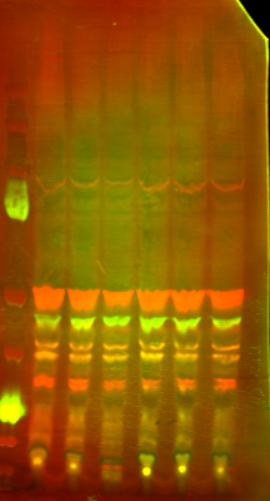


Total JNK (Red)/(P) JNK (Green)

Corresponding to Supplemental Figure 9A


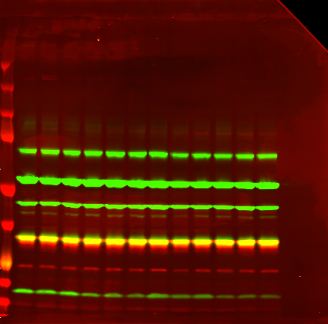


ETC protein cocktail (SDHA, ATP5A, UQCRC2, MTCO1, VDAC, NDUFB8) – Heart

Corresponding to Supplemental Figure 9B


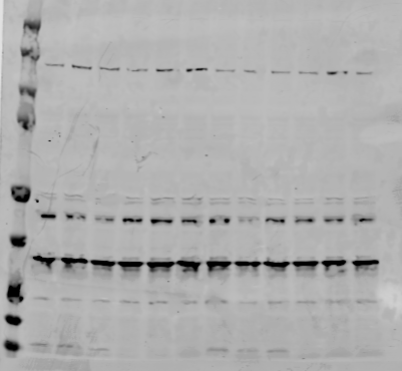


ECSIT – Heart

Corresponding to Supplemental Figure 10


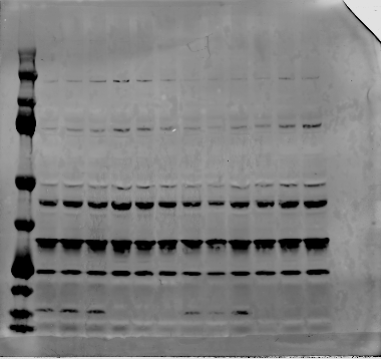


ECSIT – Heart


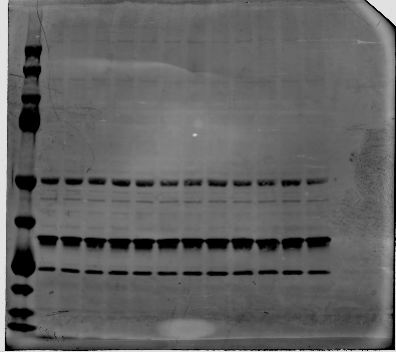


ECSIT – Brain

Corresponding to Supplemental Figure 11A


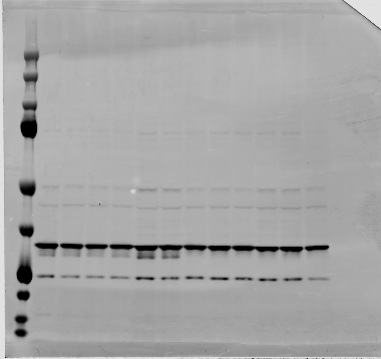


ECSIT – Kidney

Corresponding to Supplemental Figure 11B


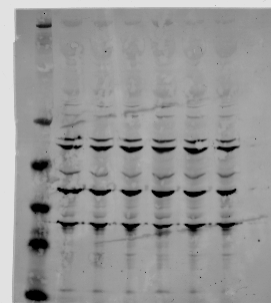


ECSIT - Liver
